# Effects of Plyometric Jump Training on Balance Performance in Healthy Participants: A Systematic Review With Meta-Analysis

**DOI:** 10.3389/fphys.2021.730945

**Published:** 2021-10-20

**Authors:** Akhilesh Kumar Ramachandran, Utkarsh Singh, Rodrigo Ramirez-Campillo, Filipe Manuel Clemente, José Afonso, Urs Granacher

**Affiliations:** ^1^Department of Physical Activity Sciences, Universidad de Los Lagos, Santiago, Chile; ^2^Exercise and Rehabilitation Sciences Laboratory, School of Physical Therapy, Faculty of Rehabilitation Sciences, Universidad Andres Bello, Santiago, Chile; ^3^Escola Superior Desporto e Lazer, Instituto Politécnico de Viana do Castelo, Rua Escola Industrial e Comercial de Nun'Álvares, Viana do Castelo, Portugal; ^4^Instituto de Telecomunicações, Delegação da Covilhã, Lisboa, Portugal; ^5^Centre for Research, Education, Innovation and Intervention in Sport, Faculty of Sport of the University of Porto, Porto, Portugal; ^6^Division of Training and Movement Sciences, University of Potsdam, Potsdam, Germany

**Keywords:** plyometric exercise, human physical conditioning, resistance training, movement, postural control, exercise

## Abstract

**Background:** Postural balance represents a fundamental movement skill for the successful performance of everyday and sport-related activities. There is ample evidence on the effectiveness of balance training on balance performance in athletic and non-athletic population. However, less is known on potential transfer effects of other training types, such as plyometric jump training (PJT) on measures of balance. Given that PJT is a highly dynamic exercise mode with various forms of jump-landing tasks, high levels of postural control are needed to successfully perform PJT exercises. Accordingly, PJT has the potential to not only improve measures of muscle strength and power but also balance.

**Objective:** To systematically review and synthetize evidence from randomized and non-randomized controlled trials regarding the effects of PJT on measures of balance in apparently healthy participants.

**Methods:** Systematic literature searches were performed in the electronic databases PubMed, Web of Science, and SCOPUS. A PICOS approach was applied to define inclusion criteria, (i) apparently healthy participants, with no restrictions on their fitness level, sex, or age, (ii) a PJT program, (iii) active controls (any sport-related activity) or specific active controls (a specific exercise type such as balance training), (iv) assessment of dynamic, static balance pre- and post-PJT, (v) randomized controlled trials and controlled trials. The methodological quality of studies was assessed using the Physiotherapy Evidence Database (PEDro) scale. This meta-analysis was computed using the inverse variance random-effects model. The significance level was set at *p* <0.05.

**Results:** The initial search retrieved 8,251 plus 23 records identified through other sources. Forty-two articles met our inclusion criteria for qualitative and 38 for quantitative analysis (1,806 participants [990 males, 816 females], age range 9–63 years). PJT interventions lasted between 4 and 36 weeks. The median PEDro score was 6 and no study had low methodological quality (≤3). The analysis revealed significant small effects of PJT on overall (dynamic and static) balance (*ES* = 0.46; 95% *CI* = 0.32–0.61; *p* < 0.001), dynamic (e.g., Y-balance test) balance (*ES* = 0.50; 95% *CI* = 0.30–0.71; *p* < 0.001), and static (e.g., flamingo balance test) balance (*ES* = 0.49; 95% *CI* = 0.31–0.67; *p* < 0.001). The moderator analyses revealed that sex and/or age did not moderate balance performance outcomes. When PJT was compared to specific active controls (i.e., participants undergoing balance training, whole body vibration training, resistance training), both PJT and alternative training methods showed similar effects on overall (dynamic and static) balance (*p* = 0.534). Specifically, when PJT was compared to balance training, both training types showed similar effects on overall (dynamic and static) balance (*p* = 0.514).

**Conclusion:** Compared to active controls, PJT showed small effects on overall balance, dynamic and static balance. Additionally, PJT produced similar balance improvements compared to other training types (i.e., balance training). Although PJT is widely used in athletic and recreational sport settings to improve athletes' physical fitness (e.g., jumping; sprinting), our systematic review with meta-analysis is novel in as much as it indicates that PJT also improves balance performance. The observed PJT-related balance enhancements were irrespective of sex and participants' age. Therefore, PJT appears to be an adequate training regime to improve balance in both, athletic and recreational settings.

## Introduction

Balance is the constant process of maintaining the center of mass vertically aligned above the base of support (feet). Postural control relies on feedforward and feedback mechanisms producing sensory information through the visual, vestibular and proprioceptive systems that are integrated and processed within the central nervous system and result in effective and coordinated neuromuscular responses (Brachman et al., 2017). There is evidence that balance performance is task specific and therefore denoted as a skill and not an ability (Haddad et al., 2013; Fong et al., 2016; Dunsky et al., 2017; Khallaf, 2020). Accordingly, it can be divided into two categories, dynamic and static balance. Dynamic balance refers to the capacity to perform a task while maintaining or regaining a stable position during locomotion (Winter et al., 1990; Kibele et al., 2015). Static balance is defined as the capacity to maintain the center of mass above the base of support with minimal movement (Hrysomallis, 2011). Balance is not only an important prerequisite for the performance of everyday tasks and the avoidance of falls but also for the successful performance of sport-specific skills in athletic populations (Boccolini et al., 2013). There is evidence that performance in bipedal static balance significantly correlated (*r* = 0.51, *p* < 0.05) with shooting accuracy. Better balance performances were noted in athletes of higher compared with lower expertise level (Mason and Pengrim, 1986). Moreover, performance in bipedal static balance was significantly associated (*r* = −0.29 to −0.45, *p* < 0.05) with shooting accuracy in novice rifle shooters (Mononen et al., 2007). In addition, performance in dynamic bipedal balance significantly correlated (*r* = 0.65, *p* < 0.05) with maximum skating speed in male ice hockey players aged ≤ 20 years (Behm et al., 2005). Besides the reported associations with performance measures, balance performance appears to be related to injury risk. Of note, high school basketball players (males and females) with balance deficits had a sevenfold increase in the risk of sustaining ankle sprains (McGuine et al., 2000). A review of the literature found that balance deficits were associated with an increased risk of injuries, including ankle sprains, muscle-tendon and ligament injuries in athletes from various sports (Brachman et al., 2017).

With reference to the principle of training specificity (Behm and Sale, 1993), balance training is usually applied if the goal is to improve balance in healthy participants (Lesinski et al., 2015a,b; Gebel et al., 2018, 2020). However, less is known on potential transfer effects of other training types (e.g., plyometric jump training [PJT]) on measures of dynamic and static balance. Commonly, PJT includes exercises that have the potential to activate large muscle groups (e.g., quadriceps). A large number of PJT drills (e.g., drop jumps) are performed in the stretch shortening cycle (SSC). The SSC is characterized by muscle-tendon lengthening during the braking phase, followed by muscle-tendon shortening during the propulsion phase (Chmielewski et al., 2006; Ramírez-Campillo et al., 2018, 2020a). The inclusion of unilateral, bilateral jump/landing drills in different directions (e.g., vertical, horizontal, lateral) and on different surfaces (e.g., stable; unstable) may provide adequate training stimuli for the somatosensory system which is responsible for controlling the body segments in space (Zech et al., 2010; Hoch et al., 2011; Peterka, 2018). Therefore, PJT exercises challenge the neuromuscular system to a high degree (Witzke and Snow, 2000; Hewett et al., 2002). Given that PJT is a highly dynamic exercise type with various forms of dynamic jump-landing tasks, high levels of postural control are needed to successfully perform PJT exercises. Accordingly, PJT has the potential to not only improve measures of muscle strength and power but also balance (Myer et al., 2006; Huang et al., 2014; Surakhamhaeng et al., 2020). Of note, PJT exercises are often incorporated in neuromuscular or multimodal training programmes which amongst other exercise types combine balance and PJT drills with the goal to improve muscle strength, balance and reduce the risk of sustaining injuries (Zemková and Hamar, 2018; Caldemeyer et al., 2020; Crossley et al., 2020). However, with reference to the relevant literature, it is not possible to elucidate the independent or isolated effect of PJT exercises within a multimodal exercise programme. With regards to PJT as single intervention programme, the available literature showed controversial effects of PJT on measures of balance in different cohorts. While Ramírez-Campillo et al. (2015b) and Makhlouf et al. (2018) reported small-to-moderate PJT effects on dynamic (i.e., Y balance test [YBT]) and static balance (i.e., stork balance test) in youth soccer players, Meszler and Váczi (2019) as well as Asadi and Arazi (2012) showed no significant effects of PJT on dynamic (i.e., star excursion balance test [SEBT]) and static balance (i.e., single-leg balance test) in youth basketball players. Accordingly, it is timely to systematically aggregate the effects of PJT on balance performance in healthy participants.

The rationale to address the proposed research question through a systematic review with meta-analysis is manifold. First, a systematic review with meta-analysis allows to aggregate the results of the available peer-reviewed literature, potentially solving the issue of controversial effects of PJT on measures of balance reported in original research. Second, a limitation of studies exploring the effects of PJT interventions is that the study outcomes are based on rather small sample sizes. Of note, a low number (i.e., <10) of participants in experimental groups is very common among PJT interventions (Ramírez-Campillo et al., 2018, 2020c). The methodological limitation of underpowered studies may partially be addressed by conducting a systematic review with meta-analysis. Third, the number of PJT-related publications in general and the number of PJT studies focusing on the effects of training on balance performance in particular has tremendously increased (25-fold) between 2000 and 2017 (Ramírez-Campillo et al., 2018). Such an increase in rate of novel publications calls for constant updates of the literature. A systematic review with meta-analysis provides an overview of the currently available literature, favoring an adequate perspective for the advancement in the field through the reporting of strengths and gaps in the literature, limitations and shortcomings related to PJT interventions. Fourth, a meta-analysis allows to aggregate the sample sizes from different studies, and may provide not only high-quality evidence, but also new insights for practitioners that help to take evidence-based decisions regarding the implementation of PJT (Murad et al., 2016).

Therefore, the primary aim of this systematic review with meta-analysis was to determine the effects of PJT compared with active controls on dynamic and static balance in apparently healthy participants. We were additionally interested in elucidating the effects of PJT on balance performance compared with specific active controls (e.g., balance training). To our knowledge, this is the first systematic review with meta-analysis that examines the effects of PJT vs. active and passive controls on balance in apparently healthy participants.

## Methods

### Procedures

A systematic literature review with meta-analysis was conducted following previously published recommendations (Liberati et al., 2009). The study was registered in PROSPERO (International Prospective Register of Systematic Reviews), an international database for systematic reviews prospectively registered by the Center for Reviews and Dissemination of the University of York (https://www.crd.york.ac.uk/prospero; CRD42021236748).

### Literature Search

Computerized literature searches were conducted in the electronic databases PubMed, Web of Science, and SCOPUS. To conduct the literature search, we considered recommendations from the two largest scoping reviews that have previously examined PJT (Ramírez-Campillo et al., 2018, 2020c). Additionally, potentially relevant keywords were collected through expert opinion. In particular, 10 distinguished experts in the field of PJT (i.e., plyometric exercise), identified through the website *Experstcape* (https://expertscape.com), were contacted to list the most appropriate key words. Organized vocabulary (i.e., Medical Subject Headings: MeSH) were also incorporated. As a result, the following key words were introduced in the electronic databases in different combinations using a Boolean search strategy with the operators “AND” and “OR”: jump, ballistic, complex, explosive, force, velocity, plyometric, stretch, shortening, and cycle.

#### Administration and Update of the Systematic Review

Electronic searches were conducted according to the specific characteristics of each electronic database search engine. After an initial search in April 2017 (Ramírez-Campillo et al., 2018), accounts were created in each of the respective databases, and through these, automatically generated email updates (PubMed alerts) were received with regards to the selected search terms. The search was refined in May 2019 (Ramírez-Campillo et al., 2020c), and updates were received daily (if available); studies were eligible for inclusion up to February 1st, 2021. The main advantage of this search approach is that it assumes that new knowledge will appear and allow improvements in sport/clinical decision-making. Indeed, the rate of PJT studies increased exponentially during the last years (Ramírez-Campillo et al., 2020c). As previously recommended (Van Der Vlist et al., 2021), we designed a protocol to extract the relevant information for this systematic review.

One of the authors (RRC) conducted the initial search and removed duplicates. Thereafter, the search results were analyzed according to the eligibility criteria. In selecting studies for inclusion, a review of all relevant titles was conducted before examination of the abstracts and full-texts. Following the formal systematic searches, additional manual searches were conducted using the authors' personal libraries and published narrative/scoping/systematic reviews and meta-analyses. Two authors (AR and US) independently screened the titles, abstracts and/or full-text versions of the retrieved studies. During the search and review process, potential discrepancies between the two authors regarding inclusion and exclusion criteria (e.g., type of control group, intervention adequacy) were resolved through consensus by including a third author (RRC).

### Inclusion and Exclusion Criteria

A PICOS (participants, intervention, comparators, outcomes, and study design) approach was used to rate studies for eligibility (Liberati et al., 2009). The respective inclusion/exclusion criteria adopted in our meta-analysis were reported in Table 1.

**Table 1:** Selection criteria used in the meta-analysis.

**Category**	**Inclusion criteria**	**Exclusion criteria**
Population	Healthy participants, with no restrictions on their fitness level, sex, or age.	Participants with health problems (e.g., injuries, recent surgery).
Intervention	A plyometric jump training programme, defined as lower body unilateral or bilateral bounds, jumps, and hops that commonly utilize a pre-stretch or countermovement stressing the stretch-shortening cycle.	Exercise interventions not involving plyometric jump training or exercise interventions involving plyometric jump training programmes representing less than 50% of the total training load when delivered in conjunction with other training interventions (e.g., high-load resistance training).
Comparator	Active or passive control group.	Absence of a control group.
Outcome	At least one measure related to balance (dynamic; static) before and after the training intervention.	Lack of baseline and/or follow-up data.
Study design	Multi-arm trials.	Single arm trials/observational studies.

Additionally, only full-text, peer-reviewed and original research were considered eligible for this meta-analysis. Books, book chapters, and congress abstracts, as well as cross-sectional papers, and training-related studies that did not focus on the effects of PJT exercises on balance performance (e.g., studies examining the effects of upper-body plyometric exercises) were excluded. We additionally excluded retrospective studies, studies in which the use of jump exercises was not clearly described, studies of which the abstract was available only, case reports, special communications, letters to the editor, invited commentaries, errata, overtraining studies, and detraining studies. In the case of detraining studies, if a training period was included prior to the detraining period, the study was considered for inclusion. Finally, in view of the potential difficulties of translating articles written in different languages—and the fact that 99.6% of the PJT literature is published in English (Ramírez-Campillo et al., 2018), only articles written in English were considered for this meta-analysis.

### Data Extraction

Means and standard deviations (SDs) of balance tests (e.g., dynamic, static, unipedal, bipedal, eyes closed, and eyes open) were used to evaluate the effects of PJT vs. active controls (any sport-related activity) or specific active controls (a specific exercise type such as balance training). For studies reporting values other than means and SDs (e.g., median, range, interquartile range, standard error values) conversion was applied as previously recommended (Wan et al., 2014; Lee et al., 2015). Different balance tasks were considered (for a full description, see Supplementary Table 1) as these may reflect different physiological and biomechanical indicators relevant to overall balance performance (Hrysomallis, 2008; Ricotti, 2011). A high intraclass correlation coefficient (≥0.8) and a low coefficient of variation (<7%) for different balance performance measures (e.g., anterior-posterior balance; medial-lateral balance; normal stance; perturbed stance; eyes open-closed; Y-balance test) has been reported previously (Ramírez-Campillo et al., 2015b); which is essential to ensure strong consistency between the analyzed studies within a meta-analysis (Liberati et al., 2009). In cases where the required data were not clearly or completely reported, the authors of the study were contacted for clarification. If no response was obtained from the authors (after two attempts), or if the authors could not provide the requested data, the study outcome was excluded from further analysis. If data were only displayed in the form of figures but not tables, the data were extracted using software to receive the relevant numbers (WebPlotDigitizer; https://apps.automeris.io/wpd/) to derive the relevant numerical data. This procedure has proven to be valid (*r* = 0.99, *p* < 0.001) (Drevon et al., 2017). Two authors (AR and US) performed data extraction independently, and discrepancies between authors (e.g., mean value for a given outcome, total number of participants in a group) were resolved through consensus with a third author (RRC).

Data were extracted from the included studies using a form created in Microsoft Excel (Microsoft Corporation, Redmond, WA, USA). Extracted data included the following information: the first author's name, study identification code (e.g., DOI), year of publication, PJT treatment description, description of the comparator (active vs. specific-active control), type of randomization, number of participants per group. We also extracted data regarding the participants' sex, age (years), body mass (kg), height (m), and previous experience with PJT. If applicable, the type and level (e.g., professional, amateur) of sport practice were also extracted. Regarding PJT programming parameters, we reported weekly frequency of training (days/week), duration (weeks), intensity level (e.g., maximal), and proxies of intensity (e.g., jumping height), jump box height (cm), number of total jumps completed during the intervention, types of jump drills performed, combination (if applicable) of PJT with another form of training type, rest time between sets (s), rest time between repetitions (s), rest time between sessions (hours), type of jumping surface (e.g., grass), type of progressive PJT overload (e.g., volume-based; technique-based), training period during the year (e.g., in-season), replaced (if applicable) portion of the regular training through PJT drills, tapering strategy (if applicable). A complete description of the PJT characteristics has been previously published (Ramírez-Campillo et al., 2018).

### Methodological Quality of the Included Studies

The Physiotherapy Evidence Database (PEDro) scale was used to assess the methodological quality of the included studies, which were rated from 0 (lowest quality) to 10 (highest quality). The validity and reliability of the PEDro scale has been established previously (Maher et al., 2003; de Morton, 2009; Yamato et al., 2017). Additionally, its agreement with other scales (e.g., Cochrane risk of bias tool) has been reported (Moseley et al., 2019). Moreover, the PEDro scale is probably the most frequently used scale in the PJT literature. Accordingly, it helps to make comparisons between meta-analyses. According to cut-off scores, the methodological quality was rated as “poor” (<4), “fair” (4–5), “good” (6–8), and “excellent” (9–10) in some sub-fields, however, it is not possible to satisfy all scale items in some areas of physiotherapy practice (Cashin and McAuley, 2020). Therefore, as outlined in previous systematic reviews in the sub-field of PJT, the methodological quality of PJT studies was interpreted using the following convention (Stojanović et al., 2017; Ramírez-Campillo et al., 2020d, 2021b): ≤3 points was considered as poor quality, 4–5 points was considered as moderate quality, and 6–10 points was considered as high quality. If trials were already rated and listed in the PEDro database, the respective scores were adopted. The methodological quality of each included study was assessed independently by two authors (AR and US), and any discrepancies between the two authors were resolved via consensus with a third author (RRC).

### Summary Measures, Synthesis of Results, and Publication Bias

Studies were meta-analytically aggregated if three or more relatively homogeneous studies were available for the same outcome measure. Effect sizes (ES; Hedge's g) were calculated for each measure of balance using means and SDs from pre- and post-tests for each dependent variable. For studies that reported standard errors (Nobre et al., 2017; Ritzmann et al., 2018), SDs were calculated by multiplying the standard error with the square root of the sample size (Lee et al., 2015). Data were standardized using post-intervention SD values. The random-effects model was used to account for differences between studies that might impact PJT effects (Deeks and Higgins, 2008; Kontopantelis et al., 2013). The ES values were presented with 95% confidence intervals (95% CIs). The ES magnitudes were interpreted using the following scale: <0.2, trivial; 0.2–0.6, small; >0.6–1.2, moderate; >1.2–2.0, large; >2.0_4.0, very large; >4.0, extremely large (Hopkins et al., 2009). In studies including more than one intervention group, the sample size of the active and specific-active control group was proportionately divided to facilitate comparisons across multiple groups (Higgins and Deeks, 2008). The impact of study heterogeneity was assessed using the I^2^ statistic, with values of <25%, 25–75%, and >75% representing low, moderate, and high levels, respectively (Higgins and Thompson, 2002). The risk of reporting bias was explored (with at least 10 studies) (Sterne et al., 2011) using the Egger's test (Egger et al., 1998), with *p* < 0.05 implying bias. To adjust for risk of reporting bias, a sensitivity analysis was conducted using the trim and fill method (Duval and Tweedie, 2000), with L_0_ as the default estimator for the number of missing studies (Shi et al., 2019). All analyses were carried out using the Comprehensive Meta-Analysis software (Version 2.0; Biostat, Englewood, NJ, USA). The level of statistical significance was set at *p* < 0.05.

### Moderator Analyses

Using a random-effects model and independent computed single factor analysis, potential sources of heterogeneity likely to influence the effects of PJT were selected a priori.

#### Subgroup Analyses

As the adaptive responses to PJT programmes may be affected by moderators such as sex (de Villarreal et al., 2009), age (Asadi et al., 2017; Moran et al., 2017a, 2019), and training background (Sáez de Villarreal et al., 2012), these factors were considered as potential moderator variables, using a categorical approach (e.g., male vs. female). Additionally, we examined the effects of PJT taking the different test situations into account (i.e., laboratory-based balance tests vs. field-based balance tests).

#### Single Factor Analyses

Single factor analyses were computed for the programmes parameter duration of intervention (number of weeks and total number of training sessions) (de Villarreal et al., 2009) and training frequency (number of weekly sessions) (de Villarreal et al., 2010) based on the reported influence of these variables on physical fitness adaptations to PJT. When appropriate, subgroup analyses and single factor analyses were divided using the median split technique (Moran et al., 2017b, 2018, 2019). The median was calculated if at least three studies provided data for a given moderator. Of note, if two experimental groups were included in a study with the same information for a given moderator (e.g., both experimental groups used a programme duration of 7 weeks), only one of the groups was considered in order to avoid an undue influence on the median calculation. In addition, to minimize heterogeneity, median values were calculated using only those studies that provided data for the outcome being analyzed. When appropriate, a logical defensible rationale was used instead of the median. A posteriori, moderator analyses were included for PJT studies that added training load to participants regular activities (e.g., sport practices) compared to those that replaced part of the regular activities with PJT.

## Results

### Study Selection

**Figure 1:**
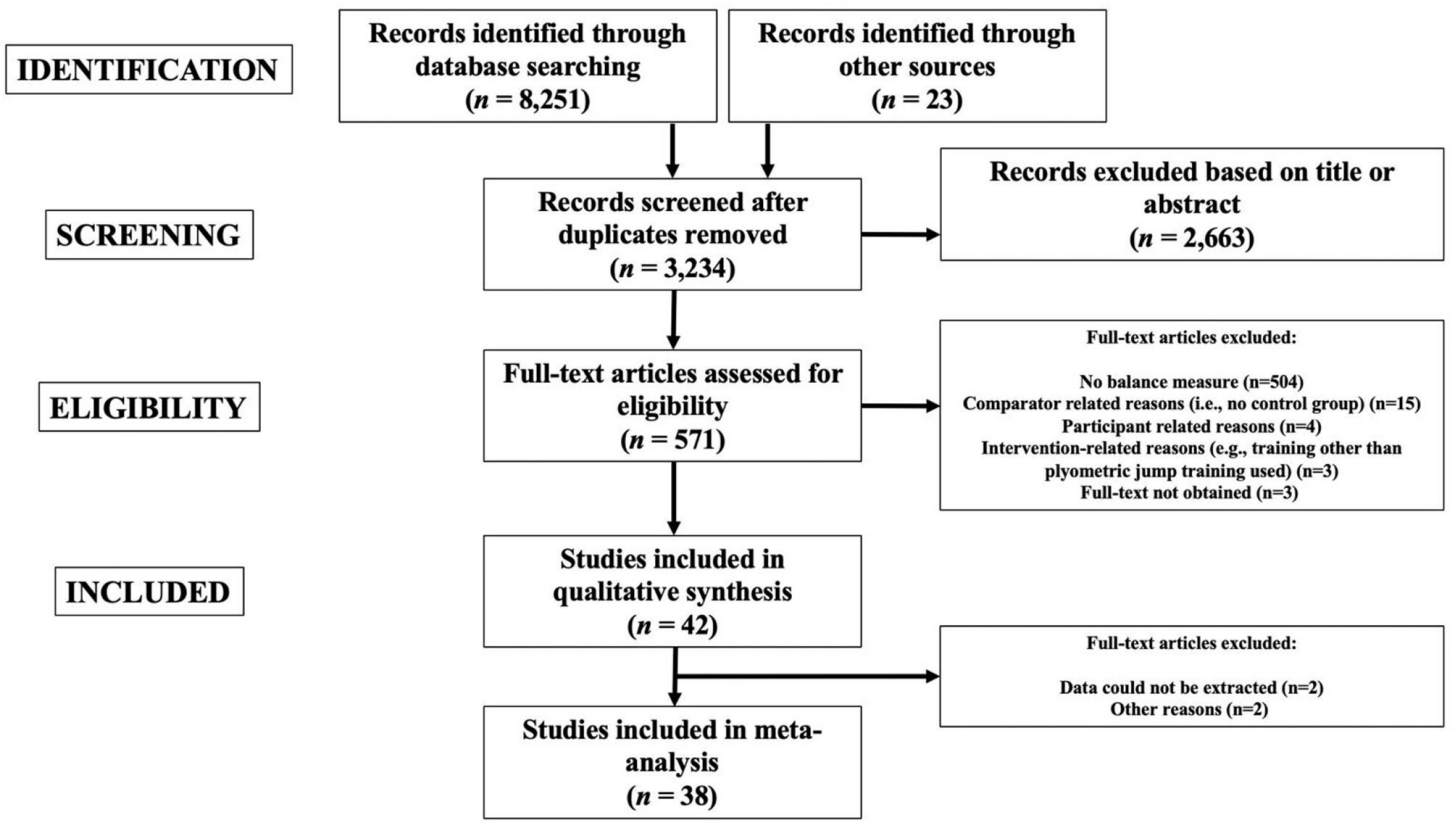
The PRISMA flow diagram.

The search process identified 8,251 studies (2,632 from PubMed; 2,612 from SCOPUS; and 3,007 from WOS). Figure 1 provides a flow chart illustrating the study selection process. Duplicate studies were removed (*n* = 5,017). After study titles and abstracts were screened, 2,663 studies were removed and 571 full-text studies were screened.

Forty-two studies were included for qualitative assessment: (Witzke and Snow, 2000; Myer et al., 2006; McLeod et al., 2009; Asadi and Arazi, 2012; Asadi, 2013; Chaouachi et al., 2014a,b; Faigenbaum et al., 2014; Huang et al., 2014; Piirainen et al., 2014; Asadi et al., 2015; Ramírez-Campillo et al., 2015a,b; Trecroci et al., 2015; Benis et al., 2016; Karadenizli, 2016; Kim and Park, 2016; Hopper et al., 2017; Nobre et al., 2017; Arabatzi, 2018; Makhlouf et al., 2018; Ritzmann et al., 2018; Alikhani et al., 2019; Cherni et al., 2019; Hammami et al., 2019a,b,c, 2020a,b,c; Jlid et al., 2019, 2020; Lovecchio et al., 2019; Meszler and Váczi, 2019; Tay et al., 2019; Akin and Kesilmiş, 2020; Bouteraa et al., 2020; Cigerci and Genc, 2020; Drouzas et al., 2020; Lee et al., 2020; Surakhamhaeng et al., 2020; Porrati-Paladino and Cuesta-Barriuso, 2021).

For meta-analysis, 38 studies were considered eligible: (Witzke and Snow, 2000; Myer et al., 2006; McLeod et al., 2009; Chaouachi et al., 2014b; Huang et al., 2014; Piirainen et al., 2014; Asadi et al., 2015; Ramírez-Campillo et al., 2015a,b; Trecroci et al., 2015; Benis et al., 2016; Karadenizli, 2016; Kim and Park, 2016; Hopper et al., 2017; Nobre et al., 2017; Arabatzi, 2018; Makhlouf et al., 2018; Ritzmann et al., 2018; Alikhani et al., 2019; Cherni et al., 2019; Hammami et al., 2019a,b,c, 2020a,b,c; Jlid et al., 2019, 2020; Lovecchio et al., 2019; Meszler and Váczi, 2019; Tay et al., 2019; Akin and Kesilmiş, 2020; Bouteraa et al., 2020; Cigerci and Genc, 2020; Drouzas et al., 2020; Lee et al., 2020; Surakhamhaeng et al., 2020; Porrati-Paladino and Cuesta-Barriuso, 2021).

Participant characteristics and PJT programmes of the included studies were detailed in Tables 2, 3, respectively.

**Table 2:** Participant's characteristics the included studies^$^.

**Study**	**Randomized**	**N**	**Sex**	**Age (years)**	**Body mass (kg)**	**Height (m)**	**SPT experience**	Fitness^*^	**Sport**
Akin and Kesilmiş (2020)	NR	20	M/F	15–19	NR	NR	NR	Normal	Taekwondo
Alikhani et al. (2019)	Yes	22	F	22	NR	NR	NR	Normal	Badminton
Arabatzi (2018)	Yes	24	M/F	9.3	36.3	1.3	No	Low	NA
Asadi and Arazi (2012)	Yes	18	M	18/ 20.4	76/60.3	1.8/1.8	No	High	Basketball
Asadi (2013)	Yes	20	M	20.2	78.5	1.82	NR	Normal	Basketball
Asadi et al. (2015)	Yes	16	M	20.1	76.4	1.85	NR	Moderate	Basketball
Benis et al. (2016)	Yes	28	F	20	62	1.72	NR	Moderate	Basketball
Bouteraa et al. (2020)	Yes	26	F	16.4	56.6	1.68	NR	Normal	Basketball
Chaouachi et al. (2014b)	Yes	26	M	13.7 /13.5	45.9/ 46.7	1.6/1.58	NR	Normal	Physical education students
Chaouachi et al. (2014a)	Yes	30	M	11	40.1	1.49	No	Normal	Judo
Cherni et al. (2019)	Yes	25	F	20.9/21	65.1/67.3	1.7/1.7	NR	High	Basketball
Cigerci and Genc (2020)	No	20	M	15.9/15.42	69.5/ 65	1.8 /1.7	NR	NCR	Basketball
Drouzas et al. (2020)^$^	Yes	45	M	9.9/10.0/10.2	39.3/36.1/38.5	1.4/1.34 /1.4	NR	Normal	Soccer
Faigenbaum et al. (2014)	Yes	40	M/F	7.6	29.5	1.24	No	Normal	NA
Hammami et al. (2019c)	Yes	41	F	13.5/13.3	42.6/42.3	1.4/1.4	NR	Moderate	Handball
Hammami et al. (2019a)	Yes	28	M	14.5/14.4	69.3	1.78	Yes	Moderate	Handball
Hammami et al. (2019b)	Yes	28	F	16.6	60.8	1.63	Yes	Moderate	Handball
Hammami et al. (2020a)	Yes	21	M	16.2/16.4/16.5	70.8/69.7/ 70.5	1.8/1.78/1.79	Yes	High	Handball
Hammami et al. (2020c)	Yes	34	F	15.8/15.8	64.2/63.0	1.66/1.67	Yes	High	Handball
Hammami et al. (2020b)	Yes	26	M	16.2/16.3/16.4	59.8/60.9/58.9^$^	1.78/1.77/1.78	NR	High	Soccer
Hopper et al. (2017)	Yes	23	F	12.1/12.3	50.7/53.3	1.64/1.63	No	Moderate	Netball
Huang et al. (2014)	Yes	20	M/F	23.20 /23.50	69.40/70.30^$^	169.30/ 170.60	NR	Normal	Mixed sports
Jlid et al. (2019)	Yes	28	M	11.8/ 11.6	36.5/ 34	1.43/1.42	NR	Moderate	Soccer
Jlid et al. (2020)	Yes	27	M	19/19	67.6/ 69.2	1.76/1.76	NR	Moderate	Soccer
Karadenizli (2016)	Yes	26	F	15.6/15.4	56.4/55.9	1.61/1.60	NR	Moderate	Handball
Kim and Park (2016)	Yes	28	F	23.5/23.2	70.2/70.3	1.78/1.76	NR	High	Volleyball
Lee et al. (2020)	Yes	14	M	22.0/23.57	69.57/66.57	1.72/1.73	NR	Moderate	Taekwondo
Lovecchio et al. (2019)	Yes	63	M	14–15	62.6/ 60.5	1.73/1.72	No	Normal	NA
Makhlouf et al. (2018)	Yes	57	M	11.1/10.98	36.9/37.22	1.45/1.45	No	Moderate	Soccer
McLeod et al. (2009)	No	62	F	15.6/16	58.9/62.3	1.7/1.71	No	Normal	Basketball
Meszler and Váczi (2019)	Yes	18	F	15.8/15.7	63.5/66.1	1.76/1.77	Yes	Moderate	Basketball
Myer et al. (2006)	Yes	19	F	15.9/15.6	61.4/66.4	1.69/1.68	Yes	Normal	Volleyball (primary sport)
Nobre et al. (2017)	Yes	59	M	9.8	41.6/43.5	1.31/1.31	No	Low	NA
Porrati-Paladino and Cuesta-Barriuso (2021)	Yes	20	M	63/56	84/77	1.76/ 1.76	No	Low	NA
Porrati-Paladino and Cuesta-Barriuso (2021)	Yes	15	F	21.11/22.38	61.83/66.16	1.63/1.62	NR	Moderate	Soccer
Ramírez-Campillo et al. (2015b)	Yes	54	M	11.0	43.5	1.46	No	Moderate	Soccer
Ramírez-Campillo et al. (2015a)	Yes	40	M	11.6	40.0	1.44	No	Moderate	Soccer
Ritzmann et al. (2018)	Yes	23	M	30	77	1.81	No	Low	NA
Surakhamhaeng et al. (2020)	Yes	20	M/F	27.70 /25.10	70.42/65.70	1.69/ 1.65	NR	Low	NA
Tay et al. (2019)	Yes	26	M/F	24.1/ 23.0	59.7/ 62.0	1.64/ 1.67	NR	Low	NA
Trecroci et al. (2015)	Yes	24	M	11.3	48.8	1.53	NR	Moderate	Soccer
Witzke and Snow (2000)	No	53	F	14.6/14.5	61/61	1.64/1.65	NR	Normal	NA

* *Fitness was classified here as it was in the recent review by Ramírez-Campillo et al., 2020c: (i) NR; (ii) high encompasses professional/elite athletes with regular enrolment in national and/or international competitions, or highly trained participants with 10 training hours per week or 6 training sessions per week and a regularly scheduled official or friendly competition; (iii) moderate encompasses non-elite/professional athletes with a regular attendance in regional and/or national competitions, between 5.09.9 training hours per week or 35 training sessions per week and a regularly scheduled official or friendly competition; and (iv) normal encompasses recreational athletes with <5 training hours per week with sporadic or no participation in competition*.

*The age, height and body mass have been mentioned for experimental/control groups*.

$ *Denotes values for studies with more than one experimental group*.

*F, female; M, male; NA, not applicable; NR, no reported; SPT, systematic experience with plyometric jump training*.

**Table 3:** Characteristics of PJT interventions the included studies.

**Authors**	**Freq**	**Dur**	**Int**	**BH (cm)**	**NTJ**	**Tply**	**Combined**	**RBS (s)**	**RBR (s)**	**RBTS (hrs)**	**Tsurf**	**PO**	**TP**	**Replace**	**Tapering**
Akin and Kesilmiş (2020)	3	6	NR	NR	NR	Mix	Yes	NR	NR	NR	NR	NP	IS	A	No
Alikhani et al. (2019)	3	6	NR	NR	NR	Mix	No	NR	NR	NR	NR	I+V+T	NA	A	No
Arabatzi (2018)	3	4	NR	NA	3,600	Mix	No	120	NA	NR	Elastic	V	NA	NA	No
Asadi and Arazi (2012)	3	8	Maximal	NA	1,188	Mix	No	60/180	NR	48	Water+land	I+V	NR	A	Yes
Asadi (2013)	2	6	Maximal	45	1,620	Mix	No	120	NR	48–120	NR	No	IS	A	No
Asadi et al. (2015)	2	6	Maximal	45	2,160	Mix	No	120	NR	72	NR	NP	PS	A	No
Benis et al. (2016)	2	8	NR	NA	>360	Mix	Yes	180	NR	24	NR	T+V	NR	R	No
Bouteraa et al. (2020)	2	8	Maximal	40–60	1,588	Mix	Yes	90	NR	48–120	NR	V+T +I	IS	R	No
Chaouachi et al. (2014b)	3	8	Maximal	NR	2,240	Mix	No	NR	NA	NR	NR	V+T	NA	NA	Yes
Chaouachi et al. (2014a)	2	12	Maximal	NR	1,080	Mix	No	180	NR	72	NR	V	NA	R	Yes
Cherni et al. (2019)	2	8	Maximal	40, 50	1,584	Mix	No	NR	NR	48	NR	I+V+T	IS	R	No
Cigerci and Genc (2020)	3	8	NR	NA	3,024	Mix	NO	180	60	48	NR	V+T	NR	A	No
Drouzas et al. (2020)	2	10	Max	10,15,20	721	Mix	No	NR	NR	48	NR	I+V+T	IS	A	Yes
Faigenbaum et al. (2014)	2	8	NR	NA	~544	Mix	Yes	NR	NR	48	NR	V	NA	NA	No
Hammami et al. (2019c)	2	9	Maximal	25, 30	630	Mix	No	90	0	48	NR	I+V+T	IS	R	No
Hammami et al. (2019a)	2	8	NR	30,40	1,536	Mix	Yes	90	NR	NR	NR	V	IS	R	No
Hammami et al. (2019b)	2	10	NR	30,40	1,920	Mix	Yes	60–120	NA	48–120	NR	No	IS	R	No
Hammami et al. (2020a)	3	7	NR	30,40	594	Mix	No	NR	NR	48	Wood	V+T	IS	R	No
Hammami et al. (2020c)	2	10	Maximal	25–40	720	Mix	No	30	0	>48	NR	I+V+T	IS	R	No
Hammami et al. (2020b)	2	10	Maximal	30, 40	960	Mix	No	30/60	NA	>48	NR	I+V+T	IS	R	No
Hopper et al. (2017)	3	6	NR	NA	1,080	Mix	Yes	NR	60	48–72	NR	I+T	IS	A	No
Huang et al. (2014)	3	6	NR	16	2,736	Mix	No	120	NA	NR	NR	T	NR	NR	No
Jlid et al. (2019)	2	8	NR	20,30	1,596	Mix	No	NR	NR	>48	NR	V+T	IS	A	No
Jlid et al. (2020)	2	6	NR	30,50	2,112	Mix	No	60	15	>48	Grass turf	V+T	IS	R	No
Karadenizli (2016)	2	10	NR	40	2,336	Mix	Yes	60–180	NR	48- 120	NR	V+T	IS	A	No
Kim and Park (2016)	3	8	NR	20, 30	3,072	Mix	No	NR	NA	NR	NR	V+I+T	NR	NR	No
Lee et al. (2020)	2	8	NR	NCR	NR	Mix	No	30	NA	48	NR	I	NR	NR	No
Lovecchio et al. (2019)	NCR	6	NR	10	NR	Mix	Yes	60	0	48–72	NR	NP	NA	NA	No
Makhlouf et al. (2018)	2	8	Maximal	NR	1,826	Mix	Balance	NR	NR	NR	NR	T+V	NR	A	Yes
McLeod et al. (2009)	2	6	NR	NA	2,380 s + 268 rep + 20 m	Mix	Yes	NR	NR	NR	NR	V+T	PS	NR	No
Meszler and Váczi (2019)	2	7	Maximal	25,35,50	1,420	Mix	No	120–300	NA	>48	NR	V+T	IS	A	Yes
Myer et al. (2006)	3	7	Maximal	NR	NR	Mix	Yes	NR	NR	24,48,96	NR	T	PS	NR	NCR
Nobre et al. (2017)	2	12	NR	10–40	1,980	Mix	NR	NR	NR	48	NR	V+ I+T	NA	NA	No
Porrati-Paladino and Cuesta-Barriuso (2021)	1–3	12	Maximal	NR	690	DJs	No	NR	NR	NA	NR	I	NA	NA	No
Porrati-Paladino and Cuesta-Barriuso (2021)	3	6	NR	NA	NCR	Mix	Yes	30/20	NA	NR	NR	I+V	NR	A	No
Ramírez-Campillo et al. (2015b)	2	6	Maximal	NA	2,160	Mix	No	60	15	48	GPT	V	IS	R	No
Ramírez-Campillo et al. (2015a)	2	6	Maximal	NA	1,610	Mix	No	60	15	>48	GPT	Yes	IS	R	No
Ritzmann et al. (2018)	5,6	8,6	NR	NA	3,744	Mix	No	NR	NR	~24	Sledge jump system	NR	NA	NA	NR
Surakhamhaeng et al. (2020)	3	6	NR	NA	2,160	Mix	No	60	NA	NR	Stable-unstable	I+T	NA	A	No
Tay et al. (2019)	2	6	NR	NA	NR	U	No	60	NA	NR	Trampoline	I+V	NA	A	No
Trecroci et al. (2015)	2	8	NR	NA	2,100	RJ	No	30–40–60	NR	NR	Artificial turf	V	IS	R	NR
Witzke and Snow (2000)	3	36	Maximal for some drills	24, 36–72	NR	Mix	Yes	NR	NR	NR	Mixed (mats, grass, concrete, wood)	V+ I+T	NA	NR	No

*A, add; APT, aquatic plyometric training; BH, box height; Dur, duration (weeks); Freq, frequency of training (days/ week); GPT, ground plyometric training; Int, intensity; IS, in-season; LPT, Land Plyometric Training; Mix, mixed PJT involved a combination of two or more of the following jumping drills, vertical, horizontal, bilateral, unilateral, repeated, non-repeated, lateral, cyclic, sport-specific (SS), slow stretch-shortening cycle, fast stretch-shortening cycle; NA, non-applicable; NP, non-progressive; NR, not reported; NTJ, number of total jumps (usually counted as jumps per each leg); PE, physical education; PJT, plyometric jump training; PO, progressive overload, in the form of either volume, intensity, type of drill, or a combination of these; PS, pre-season; R, replace; RBR, rest time between repetitions (only when the PJT programme incorporated non-repeated jumps); RBS, rest time between sets or exercise; RBTS, rest between training sessions; Repl, replace, denoting if the athletes replace some common drills from their regular training with PJT drills. If not, the PJT load was added to their regular training load; RT, resistance training; Surf, type of surface used during the intervention; T, technique; TP, training period; Tply, type of PJT drills used; Tsurf, type of surface; V, volume*.

### Methodological Appraisal of the Included Studies

According to the PEDro checklist, the median score was 6. Seven studies (4–5 points) showed moderate quality, and 35 studies were of high quality (6 points; no study scored above 6 points) (Table 4).

**Table 4:** Methodological quality of the included studies using the PEDro rating scale.

**Study name**	**Q1**	**Q2**	**Q3**	**Q4**	**Q5**	**Q6**	**Q7**	**Q8**	**Q9**	**Q10**	**Q11**	Total^*^	**Study quality**
Akin and Kesilmiş (2020)	1	0	0	0	0	0	0	1	1	1	1	4	Moderate
Alikhani et al. (2019)	1	1	0	1	0	0	0	1	1	1	1	6	High
Arabatzi (2018)	1	1	0	1	0	0	0	1	1	1	1	6	High
Asadi and Arazi (2012)	1	1	0	1	0	0	0	1	1	1	1	6	High
Asadi (2013)	1	1	0	1	0	0	0	1	1	1	1	6	High
Asadi et al. (2015)	1	1	0	1	0	0	0	1	1	1	1	6	High
Benis et al. (2016)	1	1	0	1	0	0	0	1	1	1	1	6	High
Bouteraa et al. (2020)	1	1	0	1	0	0	0	0	1	1	1	5	High
Chaouachi et al. (2014b)	1	1	0	1	0	0	0	1	1	1	1	6	High
Chaouachi et al. (2014a)	1	1	0	1	0	0	0	1	1	1	1	6	High
Cherni et al. (2019)	1	1	0	1	0	0	0	1	1	1	1	6	High
Cigerci and Genc (2020)	1	0	0	1	0	0	0	1	1	1	1	5	Moderate
Drouzas et al. (2020)	1	1	0	1	0	0	0	0	1	1	1	5	Moderate
Faigenbaum et al. (2014)	1	1	0	1	0	0	0	1	1	1	1	6	High
Hammami et al. (2019c)	1	1	0	1	0	0	0	1	1	1	1	6	High
Hammami et al. (2019a)	1	1	0	1	0	0	0	1	1	1	1	6	High
Hammami et al. (2019b)	1	1	0	1	0	0	0	1	1	1	1	6	High
Hammami et al. (2020a)	1	1	0	1	0	0	0	1	1	1	1	6	High
Hammami et al. (2020c)	1	1	0	1	0	0	0	1	1	1	1	6	High
Hammami et al. (2020b)	1	1	0	1	0	0	0	1	1	1	1	6	High
Hopper et al. (2017)	1	1	0	1	0	0	0	1	1	1	1	6	High
Huang et al. (2014)	1	1	0	1	0	0	0	1	1	1	1	6	High
Jlid et al. (2019)	1	1	0	1	0	0	0	1	1	1	1	6	High
Jlid et al. (2020)	1	1	0	1	0	0	0	1	1	1	1	6	High
Karadenizli (2016)	1	1	0	1	0	0	0	1	1	1	1	6	High
Kim and Park (2016)	1	1	0	1	0	0	0	1	1	1	1	6	High
Lee et al. (2020)	1	1	0	1	0	0	0	1	1	1	1	6	High
Lovecchio et al. (2019)	1	1	0	1	0	0	0	1	1	1	1	6	High
Makhlouf et al. (2018)	1	1	0	1	0	0	0	1	1	1	1	6	High
McLeod et al. (2009)	1	0	0	1	0	0	1	0	1	1	1	5	Moderate
Meszler and Váczi (2019)	1	1	0	1	0	0	0	1	1	1	1	6	High
Myer et al. (2006)	1	1	0	1	0	0	0	0	1	1	1	5	Moderate
Nobre et al. (2017)	1	1	0	1	0	0	0	1	1	1	1	6	High
Porrati-Paladino and Cuesta-Barriuso (2021)	1	1	0	1	0	0	0	1	1	1	1	6	High
Porrati-Paladino and Cuesta-Barriuso (2021)	1	1	0	1	0	0	0	0	1	1	1	5	Moderate
Ramírez-Campillo et al. (2015b)	1	1	0	1	0	0	0	1	1	1	1	6	High
Ramírez-Campillo et al. (2015a)	1	1	0	1	0	0	0	1	1	1	1	6	High
Ritzmann et al. (2018)	1	1	0	1	0	0	0	1	1	1	1	6	High
Surakhamhaeng et al. (2020)	1	1	0	1	0	0	0	1	1	1	1	6	High
Tay et al. (2019)	1	1	0	1	0	0	0	1	1	1	1	6	High
Trecroci et al. (2015)	1	1	0	1	0	0	0	1	1	1	1	6	High
Witzke and Snow (2000)	1	0	0	0	0	0	0	1	1	1	1	4	Moderate

*A detailed explanation for each PEDro scale item can be accessed at https://www.pedro.org.au/english/downloads/pedro-scale*.

* *From a possible maximal punctuation of 10*.

### Study Characteristics

A total of 1,061 participants were analyzed in the intervention arms and 745 participants were assessed in the active control groups; of those *n* = 142 were specific-active controls (7 groups). The duration of the training programmes in the intervention and control groups ranged from 4 to 36 weeks and the frequency of weekly training sessions ranged from 1 to 3 in most studies, except for Ritzmann et al. (2018), in which 5–6 sessions/week were conducted. Of the 42 studies, 19 included PJT interventions performed at maximal intensity, while the remaining studies did not provide any details regarding PJT intensity.

Regarding the reporting of adverse health effects of PJT, 12 studies (Myer et al., 2006; Ramírez-Campillo et al., 2015a,b; Benis et al., 2016; Nobre et al., 2017; Makhlouf et al., 2018; Hammami et al., 2019b,c, 2020a,b,c; Jlid et al., 2019) reported no adverse health events due to PJT. Two studies (Kim and Park, 2016; Porrati-Paladino and Cuesta-Barriuso, 2021) reported drop outs due to injuries. While in the study of Porrati-Paladino and Cuesta-Barriuso (2021), the injuries were unrelated to PJT, there is no such information in the study of Kim and Park (2016). The remaining 24 included studies failed to report specific information regarding adverse health effects.

A total of 274 balance measures were applied among the 38 included studies (7.2 measurements per study). From the 38 studies which were considered eligible for this meta-analysis, 29 studies used tests of dynamic balance (e.g., Y balance test) and 24 studies tests of static balance (e.g., flamingo balance test). If several tests were included in one study which all deemed to measure static or dynamic balance, Cochrane-based decision rules were applied (Supplementary File 1).

Concerning dynamic balance, 169 tests were applied among 29 studies (5.8 measurements per study). The dynamic tests were further divided into field-based tests (YBT, 14 studies; SEBT, 8 studies; backward walk test, 1 study; dynamic balance error scoring system test [BESS], 1 study), and laboratory-based dynamic balance test (6 studies; mainly involving subjects standing on unstable surfaces over balance and force-platforms).

A total of 105 static measurements were applied among 24 studies (4.4 measurements per study). The static tests were further divided into field-based tests (standing stork test; flamingo test; static BESS test; Romberg test; 14 studies) and laboratory-based static tests (10 studies; mainly involving subjects standing on stable surfaces over balance and force-platforms).

The balance measurement and assessment protocols for each of the included studies in the meta-analysis was detailed in Supplementary Table 1.

### Results From Meta-Analysis

#### Overall Static and Dynamic Balance

**Figure 2:**
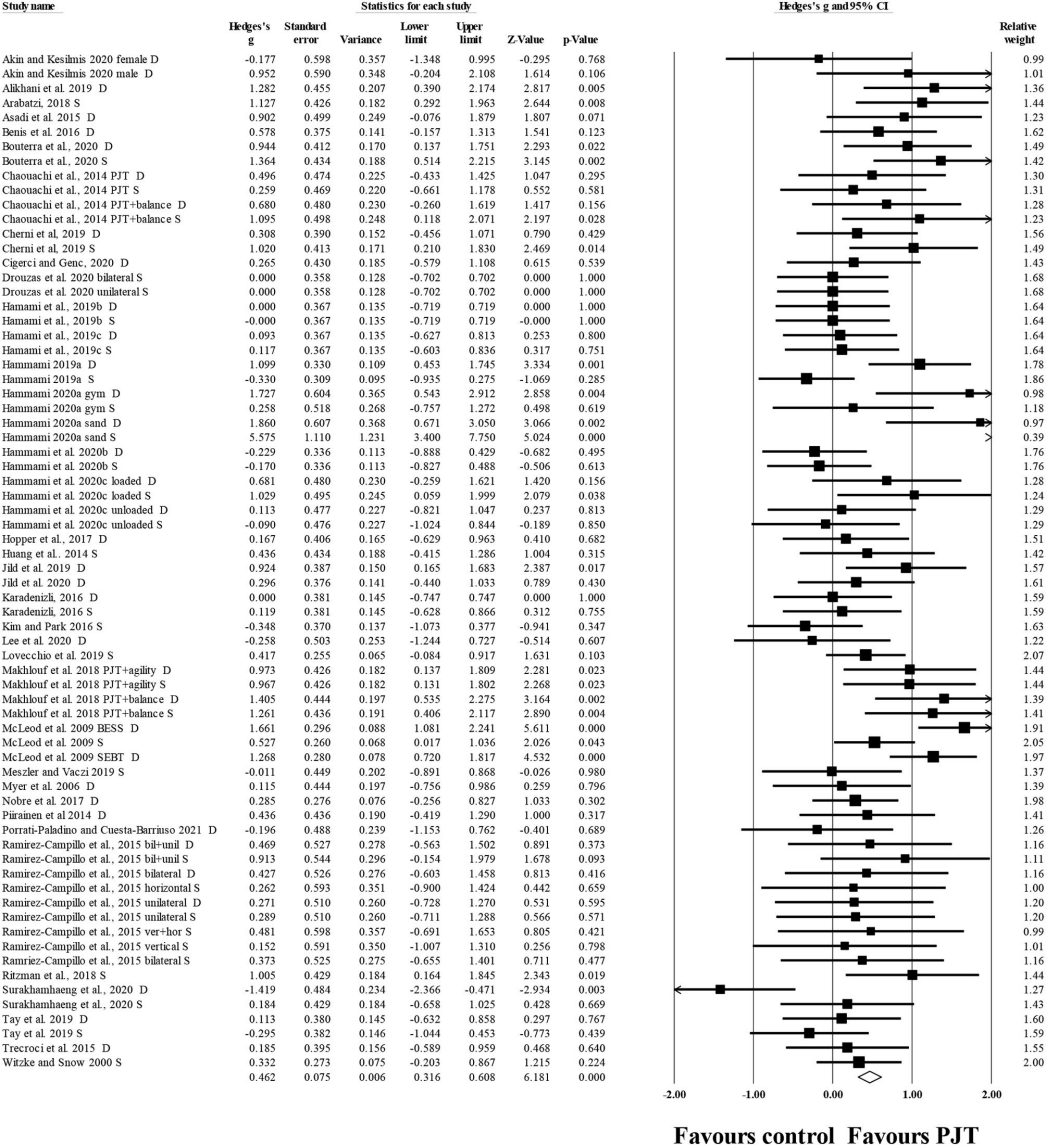
Forest plot of changes in overall balance (i.e., all dynamic and static tests included) in participants that completed a plyometric jump training (PJT) program compared to participants allocated as controls. Values shown are effect sizes (Hedges's g) with 95% confidence intervals (CI). The size of the plotted squares reflects the statistical weight of each study. The diamond reflects the overall result.

Thirty-eight studies (*n* = 1,156; 48 experimental groups, 32 active control groups, 7 specific-active control groups) provided balance data including dynamic and static tests. There was a significant small effect of PJT on overall balance compared to baseline performance (i.e., pre PJT intervention) (*ES* = 0.46; 95% *CI* = 0.32–0.61; *p* < 0.001; *I*^2^= 55.2%; Egger's test *p* = 0.152; Figure 2).

#### Dynamic Balance

**Figure 3:**
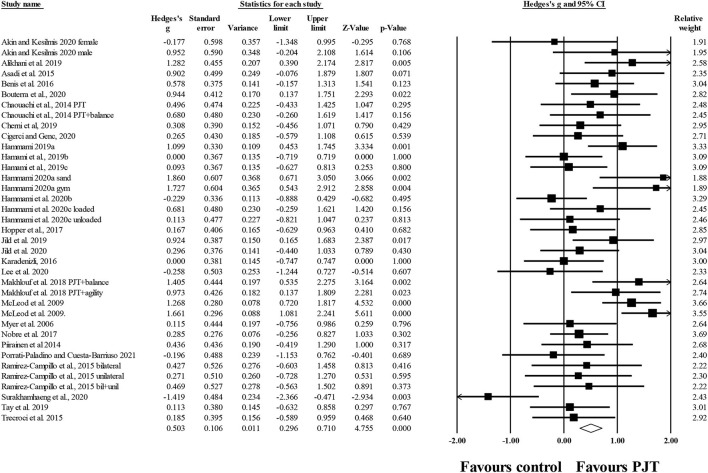
Forest plot of changes in overall dynamic balance (i.e., all dynamic tests included) in participants that completed a plyometric jump training (PJT) program compared to participants allocated as controls. Values shown are effect sizes (Hedges's g) with 95% confidence intervals (CI). The size of the plotted squares reflects the statistical weight of each study. The diamond reflects the overall result.

Twenty-nine studies provided information of PJT on dynamic balance (i.e., overall, all dynamic tests included), involving 37 experimental and 31 control groups (*n* = 933; 24 active and 7 specific-active). There was a significant small effect of PJT on dynamic balance compared to baseline performance (*ES* = 0.50; 95% *CI* = 0.30–0.71; *p* < 0.001; *I*^2^ = 57.0%; Egger's test *p* = 0.459; Figure 3).

#### Field-Based Tests of Dynamic Balance

**Figure 4:**
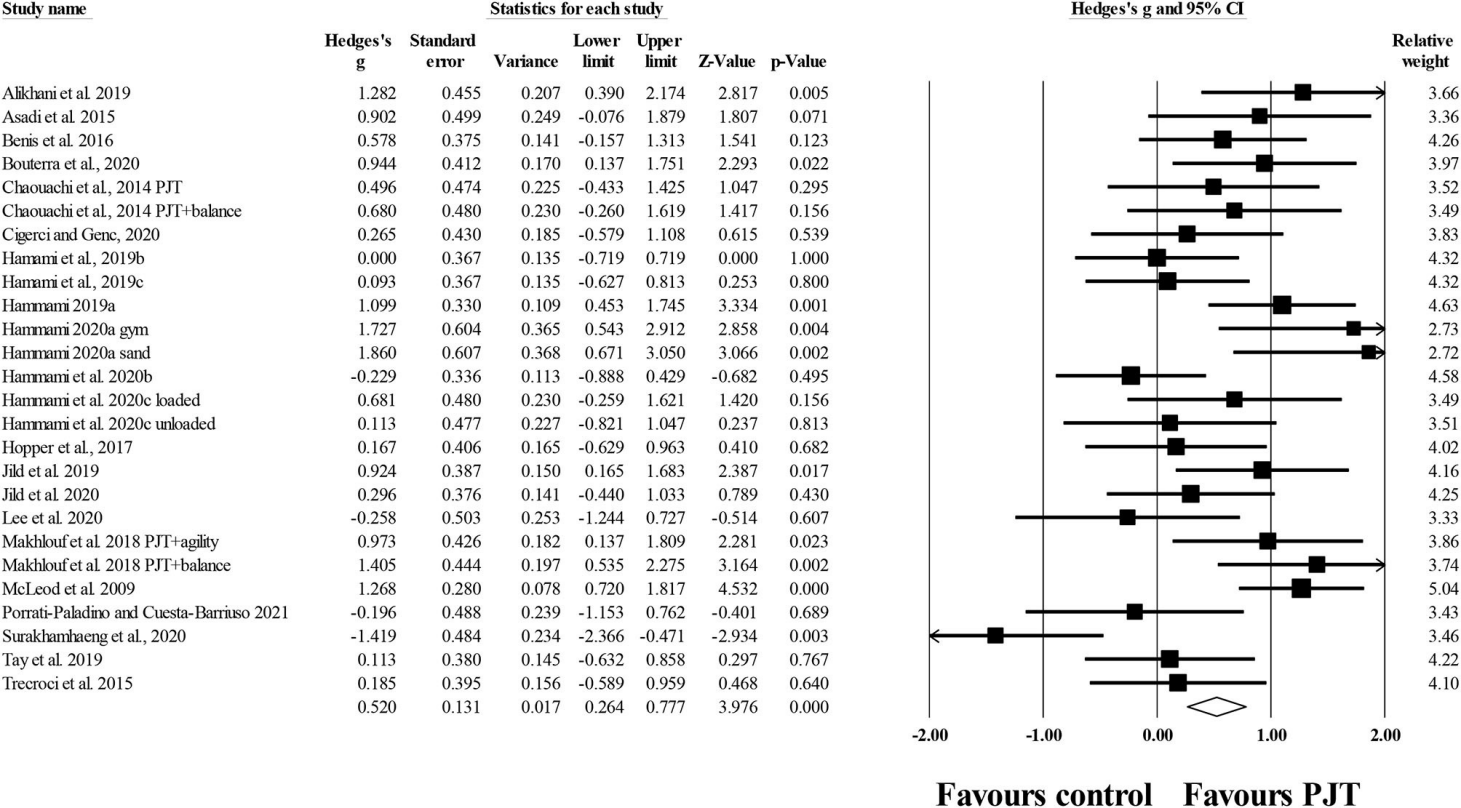
Forest plot of changes in dynamic balance, measured through field-based tests, in participants that completed a plyometric jump training (PJT) program compared to participants allocated as controls. Values shown are effect sizes (Hedges's g) with 95% confidence intervals (CI). The size of the plotted squares reflects the statistical weight of each study. The diamond reflects the overall result.

Twenty-two studies provided data for field-based tests of dynamic balance, involving 26 experimental and 22 control groups (*n* = 648; 18 active and 4 specific-active). There was a significant small effect of PJT on field-based tests of dynamic balance compared to baseline performance (*ES* = 0.52; 95% *CI* = 0.26–0.78; *p* < 0.001; *I*^2^ = 60.3%; Egger's test *p* = 0.944; Figure 4).

#### Laboratory-Based Tests of Dynamic Balance

**Figure 5:**
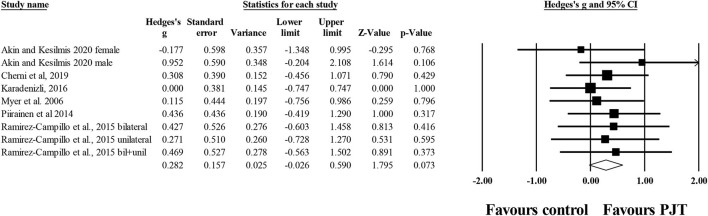
Forest plot of changes in dynamic balance, measured through laboratory-based equipment, in participants that completed a plyometric jump training (PJT) program compared to participants allocated as controls. Values shown are effect sizes (Hedges's g) with 95% confidence intervals (CI). The size of the plotted squares reflects the statistical weight of each study. The diamond reflects the overall result.

Six studies provided data for dynamic balance, measured through laboratory-based equipment, involving 9 experimental and 7 control groups (*n* = 164; 5 active and two specific-active). There was a non-significant small effect of PJT on dynamic balance, measured through laboratory-based equipment vs. baseline performance (*ES* = 0.28; 95% *CI* = −0.03–0.59; *p* = 0.073; *I*^2^ = 0.0%; Egger's test *p* = 0.346; Figure 5).

#### Static Balance

**Figure 6:**
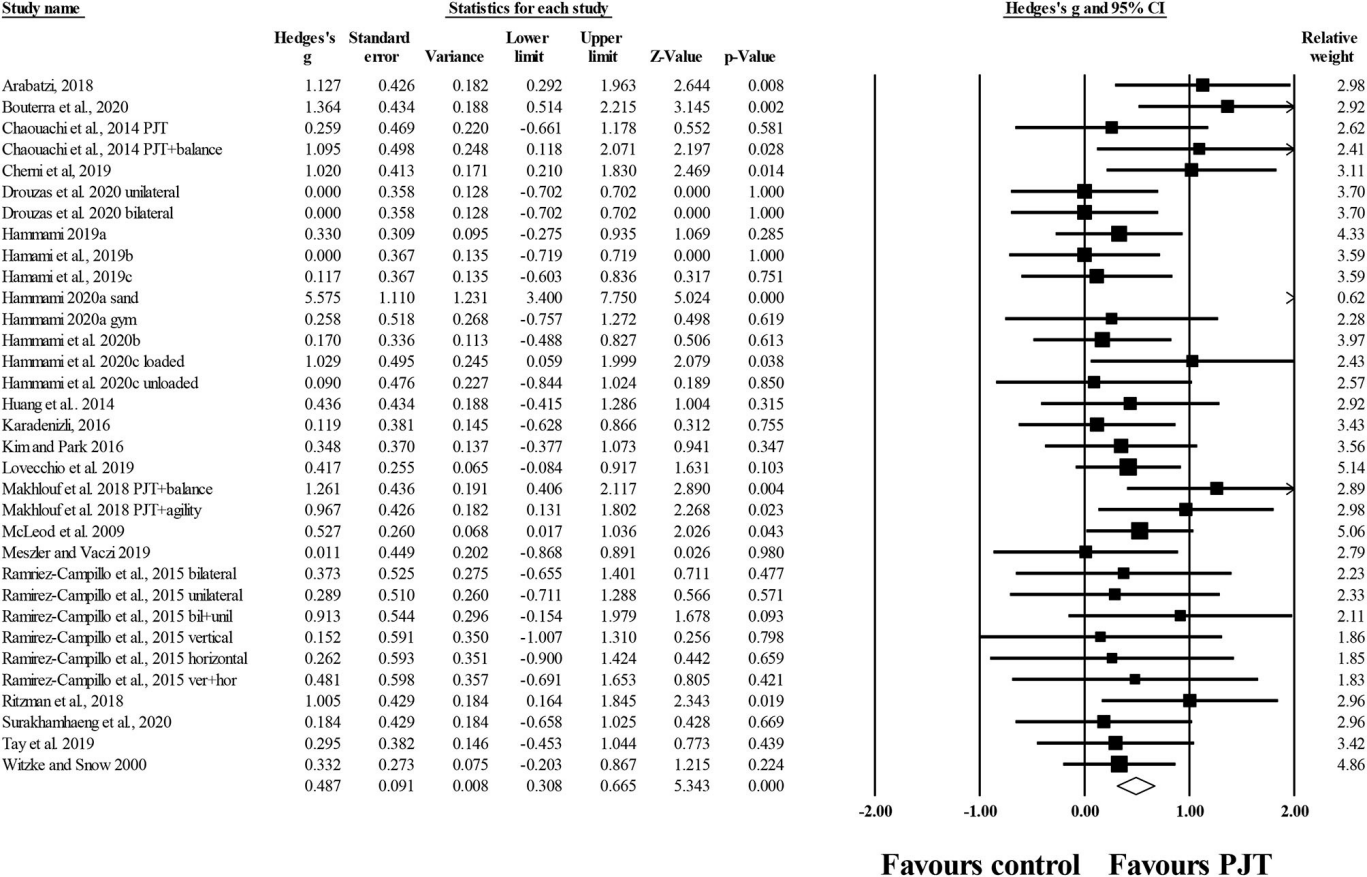
Forest plot of changes in overall static balance (i.e., all static tests included) in participants that completed a plyometric jump training (PJT) program compared to participants allocated as controls. Values shown are effect sizes (Hedges's g) with 95% confidence intervals (CI). The size of the plotted squares reflects the statistical weight of each study. The diamond reflects the overall result.

Twenty-four studies provided data on static balance tests involving 33 experimental and 24 control groups (*n*= 873; 21 active and 3 specific-active). There was a significant small effect of PJT on static balance compared to baseline performance (*ES* = 0.49; 95% *CI* = 0.31–0.67; *p* < 0.001; *I*^2^ = 37.1%; Egger's test *p* = 0.012, with adjusted values equal to the observed values after the application of the Duval and Tweedie's trim and fill method; Figure 6).

#### Field-Based Tests of Static Balance

**Figure 7:**
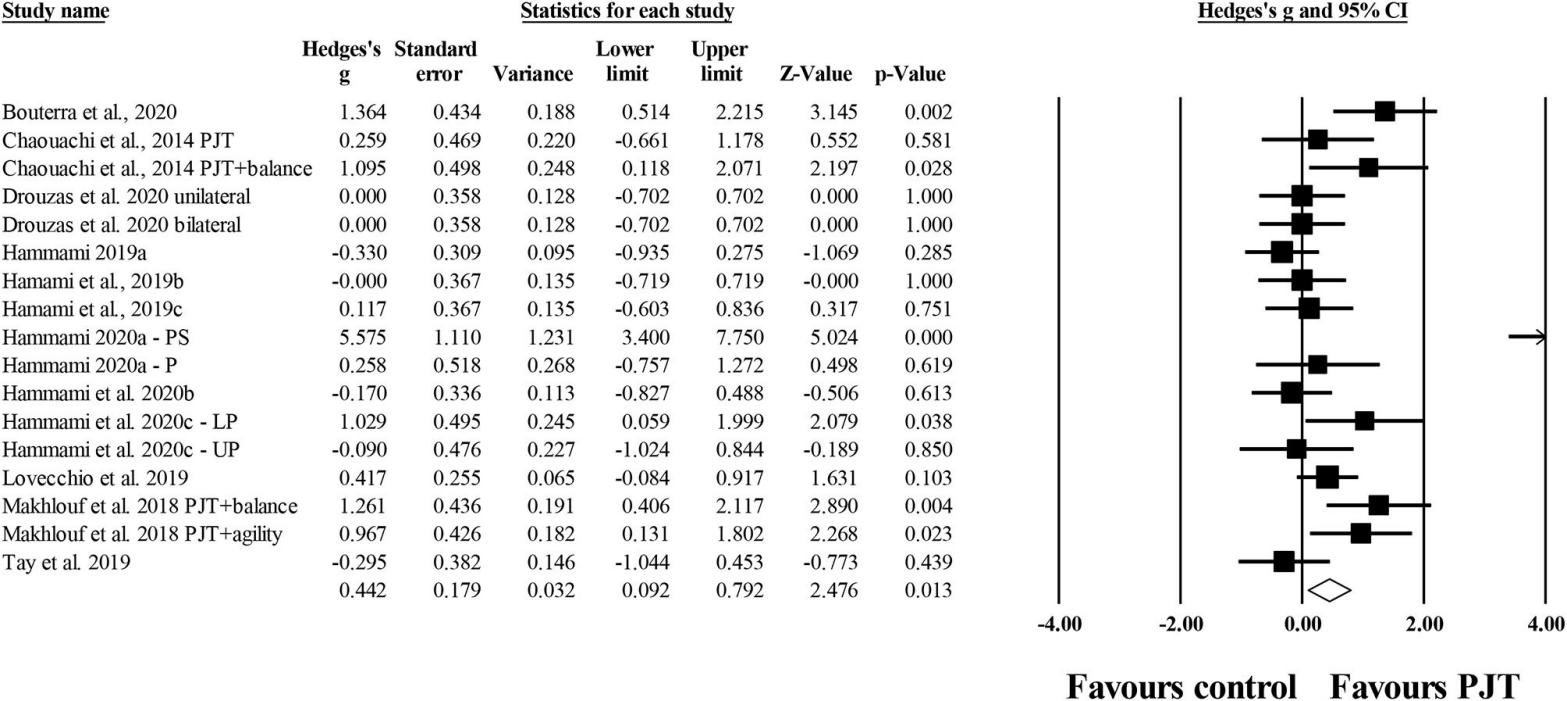
Forest plot of changes in static balance, measured through field-based tests, in participants that completed a plyometric jump training (PJT) program compared to participants allocated as controls. Values shown are effect sizes (Hedges's g) with 95% confidence intervals (CI). The size of the plotted squares reflects the statistical weight of each study. The diamond reflects the overall result.

Twelve studies provided data for field-based static balance tests involving 17 experimental and 12 control groups (*n* = 414; 11 active and 1 specific-active). There was a significant small effect of PJT on static balance compared to baseline performance (*ES* = 0.44; 95% *CI* = 0.09–0.79; *p* = 0.013; *I*^2^= 69.5%; Egger's test *p* = 0.003, with adjusted values similar to the observed values after the application of the Duval and Tweedie's trim and fill method; Figure 7).

#### Laboratory-Based Tests of Static Balance

**Figure 8:**
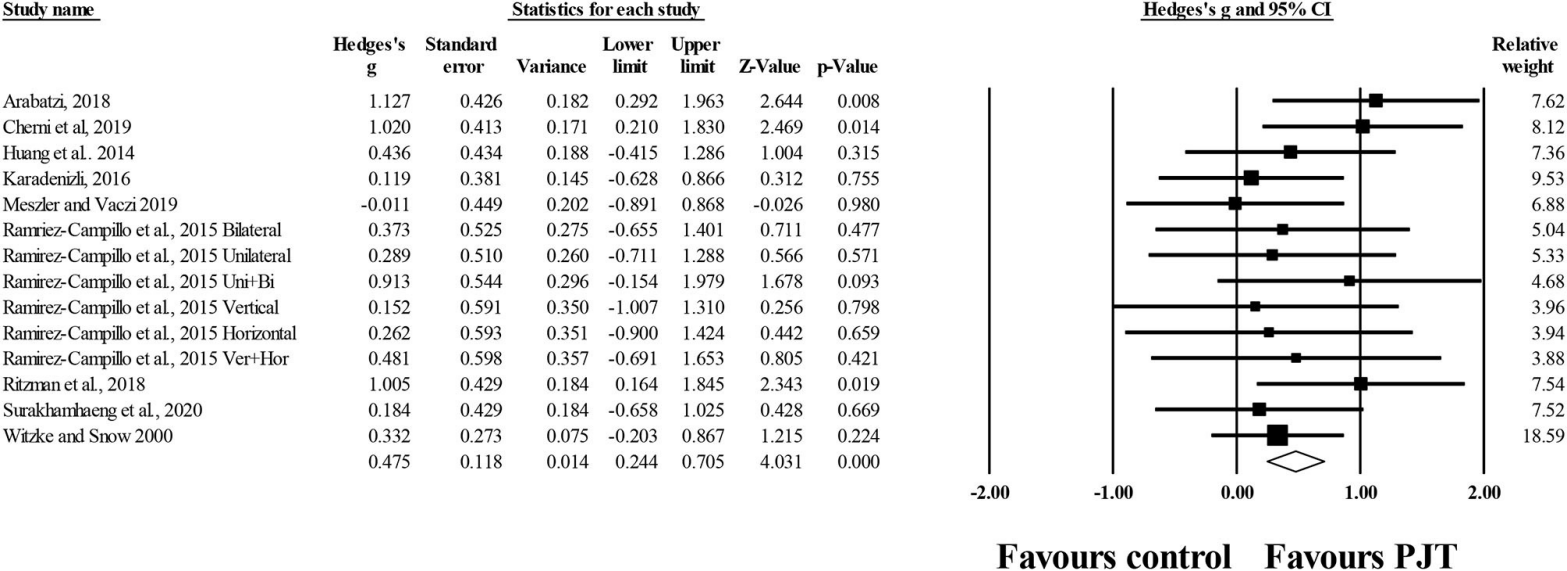
Forest plot of changes in static balance, measured through laboratory-based equipment, in participants that completed a plyometric jump training (PJT) program compared to participants allocated as controls. Values shown are effect sizes (Hedges's g) with 95% confidence intervals (CI). The size of the plotted squares reflects the statistical weight of each study. The diamond reflects the overall result.

Ten studies provided data for laboratory-based static balance tests involving 14 experimental and 10 control groups (*n* = 303; nine active and one specific-active). There was a significant small effect of PJT on laboratory-based static balance tests vs. baseline performance (*ES* = 0.48; 95% *CI* = 0.24–0.71; *p* < 0.001; *I*^2^ = 0.0%; Egger's test *p* = 0.856; Figure 8).

#### Plyometric Jump Training Compared to Specific-Active Controls

Seven studies compared the effects of PJT vs. specific-active controls (balance training, 3 studies; whole body vibration training, 1 study; resistance training, 3 studies) on measures of dynamic and static balance. The comparison involved 7 experimental (*n* = 69) and 7 specific-active controls (*n* = 73). Both PJT and specific-active controls showed similar balance effects pre- and post- PJT intervention (*p* = 0.534 between conditions). If the effects of single mode balance training (3 studies) were contrasted with PJT, similar results were obtained (*p* = 0.510).

#### Replacement of Regular Training With Plyometric Jump Training

When studies added PJT to participant's regular training activities, small significant overall balance improvement were noted post-PJT intervention (*ES* = 0.35; *p* = 0.014), similar to those studies that replaced part of the regular training with PJT (*ES* = 0.47; *p* < 0.001). No statistically significant difference was found (*p* = 0.494).

### Moderator Analyses

Using a random-effects model, potential sources of heterogeneity likely to influence the effects of PJT were analyzed. A short summary of the outcomes is provided below. More detailed information can be found in Supplementary File 2.

#### Sub-group Analyses

Participant's sex and age did not moderate the effects of PJT on measures of balance. Additionally, if laboratory-based (*ES* = 0.41) vs. field-based (*ES* = 0.49) tests of balance were taken into consideration, the type of test did not moderate the PJT effects on measures of dynamic and static balance (*p* = 0.574). However, significant (*p* = 0.047) difference in the magnitude of effects sizes were found for overall balance (*p* = 0.047) with the largest effects in basketball (*ES* = 0.83), followed by soccer (*ES* = 0.48), handball (*ES* = 0.43), and non-athletic populations (*ES* = 0.20).

#### Single Factor Analyses of Programming Parameters

With regards to weekly training frequency, significantly greater effects (*p* = 0.044) were found for PJT programmes with a frequency of ≤2 sessions/week (*ES* = 0.89) compared with to >2 weekly sessions (*ES* = 0.05) on measures of dynamic balance. No other programming parameter moderated the observed effects of PJT on measures of balance.

## Discussion

This meta-analysis aimed to examine the effect of PJT on balance performance. Overall findings of this study revealed that, compared to active controls, PJT showed moderate effects on overall balance, dynamic and static balance, irrespective of participants' sex or age. Additionally, PJT produced similar balance improvements compared to other training types (e.g., balance training).

### Comparison of PJT With Balance Training

Balance training is applied if the goal is to improve dynamic and static balance in athletes and non-athletic populations (Behm and Sale, 1993). Previous meta-analyses have shown that balance training is effective in improving balance in healthy youth aged 9–19 years (ES range: 0.61–1.03) (Gebel et al., 2018, 2020), healthy young adults aged 16–40 years (ES range: 0.32–1.29) (Lesinski et al., 2015a) and healthy older adults aged ≥65 years (ES range: 0.44–1.93) (Lesinski et al., 2015b). Our meta-analysis indicated that besides balance training, PJT is also an effective exercise type to improve balance in healthy participants. However, the observed balance improvements following PJT were somewhat lower (*ES* < 0.52) compared to the magnitudes of balance improvement in the aforementioned balance training studies. The impact of a training programme on performance depends on the type of exercise administered during the training sessions. This is well in line with the principle of training specificity (Behm and Sale, 1993). Of note, our meta-analysis revealed that when PJT was compared with balance training, both training types induced similar balance adaptations (*p* = 0.510 between training methods). Plyometric jump training has the potential to improve muscle strength, power, and balance through primarily neural adaptations (Lee et al., 2020). Compared to other training types, PJT exercises involve the braking (eccentric) and propulsion (concentric) phases performed in the SSC which, when applied over longer periods, improve muscle strength, power, and speed (Komi and Gollhofer, 1997; Taube et al., 2012). Since high levels of muscle power are crucial to maintain or regain balance during everyday (e.g., stumbling) and sport-related activities (e.g., jump-landing tasks) (Vetrovsky et al., 2019), PJT appears to be an adequate means to promote balance through increases in muscle power (Vetrovsky et al., 2019). A limitation is that only three studies were available for direct meta-analytical comparison between PJT and balance training. Further research needs to be conducted which contrasts different training types (including balance training) with PJT to better understand this subject.

### Potential Moderators of PJT-Related Effects

Previous studies suggested a maturational threshold that moderates responses (i.e., jumping) to PJT in youth (de Villarreal et al., 2009; Moran et al., 2017a). However, for balance-related outcomes, our analyses revealed no effect of sex (or age) on dynamic and static balance after PJT. Therefore, PJT seems effective to improve balance across the maturational spectrum, taking participants' fitness levels and motor competence into consideration. However, future studies should elucidate whether maturation, sex or training experience interact with PJT and balance outcomes. In addition, we found a comparable effect of <2 vs. >2 weekly PJT sessions on most of the analyzed balance measures. Moreover, the SEBT improved more after PJT with a frequency of ≤2 sessions per week (*ES* = 0.89) as compared to >2 sessions per week (*ES* = 0.05), which may be related to the greater number of including unilateral, bilateral, vertical and horizontal drills during training sessions with a lower overall PJT frequency. This higher jump drill density during a single session may provide a more demanding balance stimulus (Ramírez-Campillo et al., 2015a,b). In addition, a lower weekly PJT frequency may allow players to devote more time to other aspects of their physical conditioning (Ramírez-Campillo et al., 2020b). These findings also emphasize the importance of PJT contents rather than PJT volume (Ramírez-Campillo et al., 2015b). Therefore, rather than PJT frequency, other PJT programming parameters such as the type of jump exercise might have a greater impact on balance adaptations. For example, insufficient PJT volume, intensity, or a combination of both, may mask even greater balance improvements due to PJT with higher training frequency (Vetrovsky et al., 2019).” Additionally, the PJT studies that added training load to participant's regular activities showed a small significant overall balance improvement (*ES* = 0.35), similar to those studies that replaced parts of their regular training with PJT (*ES* = 0.47). Therefore, further research concerning the influence of PJT dosage on balance is required to better understand this subject.

Our analysis found greater PJT-related balance improvements in athletes (ES range: 0.43–0.84) compared to non-athletic populations (ES: 0.20). A qualitative analysis of the studies conducted with non-athletic populations revealed that the included participants were aged 9–65 years. Training-induced improvements maybe diminished in older compared with younger adults. The studies that examined athletic populations had an age range of 9–23 years. The differences in age between athletic and non-athletic populations could be responsible for the different effect sizes. These findings suggests that athletes may be physiologically predisposed to greater balance-related adaptations with latter training interventions (Lauber et al., 2021). This finding is similar to the greater balance improvement noted in athletes compared to non-athletic populations after balance training (Lesinski et al., 2015a). To be able to perform various multidirectional movements such as jumping, linear sprints and change of direction tasks, balance is an essential prerequisite for sport-specific performance in sports such as soccer, basketball and handball (Ramírez-Campillo et al., 2015b, 2020b). Athletes regularly perform these movements during training practice as well as matches. With reference to their training history, athletes may have developed better kinesthetic awareness and body control compared to non-athletes (Davlin, 2004). This, in turn, may have resulted in larger PJT effects on overall balance. This is in line with previous literature, suggesting that improved kinesthetic awareness and body control through the application of balance training before PJT, would induce greater improvements in balance performance after PJT (Hammami et al., 2016).

### Adverse Health Effects Derived From PJT Interventions

There were no intervention-related injuries reported in the studies included in our meta-analysis. The relative safety of PJT programmes has been previously reported (Mason and Pengrim, 1986; Markovic and Mikulic, 2010; Ramírez-Campillo et al., 2020c). PJT interventions may actually reduce the risk of injury, provided they are adequately programmed and performed under supervision (Rössler et al., 2014, 2016). However, this type of training should not be recommended to unfit athletes or adults with low strength/power levels, poor motor competency and an inability to decelerate their body mass during landing tasks (Ramírez-Campillo et al., 2020c). There is ample evidence in the literature regarding the risk of higher PJT volumes on risk of injury, especially in female athletes (Brumitt et al., 2016, 2018). Given that a reduction in PJT volume correlate with reduced overload-induced inflammation from large eccentric loads (Choi, 2014; Fransz et al., 2018), lower PJT volumes appear to be better suited to improve overall balance. While none of the included studies reported adverse health effects, 23 studies did not report participants' previous experience with PJT. Moreover, there was no information regarding the movement quality during plyometric jump drills and progressive overload in any of the included studies. Even though a potential relation has been reported previously between movement competency and PJT progression (Lloyd et al., 2011, 2016; Meylan et al., 2014) along with some factors potentially associated with the safety of PJT drills (Davies et al., 2015), conclusive evidence is still lacking. Further, there is also paucity in regards of the exact dosage and progression of programming parameters in PJT (Chmielewski et al., 2006) and in terms of the use of adequate proxies of PJT (Ebben, 2007; Ramírez-Campillo et al., 2018). Therefore, further research should be conducted to receive a better understanding of this topic. Further, 24 of the included studies in this meta-analysis failed to report specific information regarding adverse health effects. This reflects a larger problem in sports sciences and produces unbalanced accounts, as authors report the main effects, but not the adverse health effects.

### Methodological Quality

Even though all included studies were of moderate-to-high quality, none of the studies scored more than 6 points on the PEDro scale. According to the available evidence in the literature, previous systematic PJT review (Bedoya et al., 2015; Stojanović et al., 2017) have rated the published studies in this area as medium quality using the PEDro scale. A few potential reasons for this could be due to the difficulties in conducting studies that include blinding of participants and therapists. A recent PJT scoping review of Ramírez-Campillo et al. (2020c) highlighted several methodological shortcomings based on the analysis of 420 studies, with the most prominent issue being an incomplete description of training intervention characteristics, and difficulties with the randomization process and the incorporation of control groups, particularly among highly-trained athletes. Even though the included studies in our meta-analysis generally reported a clear description of the training interventions, a few key programming parameters such as rest between sets, repetitions and training intensity were not clearly reported in a few studies. Future PJT studies should try to provide a better description of all the parameters that were considered while designing the training programme to improve overall methodological quality.

### Potential Physiological Mechanisms Responsible for Balance Improvement After PJT

Our results revealed small (up to *ES* = 0.56) PJT-related improvements in measures balance compared to active controls. Compared to PJT-related improvements of other physical fitness and athletic traits (e.g., linear sprint speed; vertical jump performance; small to large *ES* = 0.60–2.24) (Shi et al., 2019; Ramírez-Campillo et al., 2020a,d), the observed balance improvements were small but meaningful and achieved the level of statistical significance. These findings are in agreement with previous studies, where PJT improved balance by promoting anticipatory postural adjustments (Gantchev and Dimitrova, 1996). Repeated exposure to balance challenges during PJT (e.g., landings) favors proactive or feedforward adjustments that appropriately activate muscles before landing (Marigold and Patla, 2002; Paillard et al., 2006). The sensitivity of the afferent feedback loops can also be improved using PJT (Borghuis et al., 2008). The PJT programmes in our meta-analysis combined unilateral, bilateral, horizontal and vertical jumping exercises which is in line with the requirements of multiple direction actions required in different sports (e.g., soccer). The improvements can also be attributed to reduced agonist-antagonist co-activation of lower-limbs muscles (Lloyd, 2001) or changes in proprioception and neuromuscular control (Hewett et al., 2002). PJT induces different neuromuscular adaptations potentially related to postural control (Ramírez-Campillo et al., 2021a), such as an increased neural drive, improved inter-muscular coordination, changes in muscle size and architecture, and/or changes in single-fiber mechanics, as well as changes in muscle-tendon mechanical-stiffness (Markovic and Mikulic, 2010). Some of these adaptations may improve balance. However, the discussion of mechanisms underlying improved balance after PJT remain speculative in our meta-analysis, with further empirical research needed to elucidate such mechanisms.

## Limitations

There are a few limitations of our study that should be emphasized. First, additional analyses regarding PJT frequency, duration, and total sessions could not be performed for all balance performance measures due to limited availability of studies (less than three) for at least one programming parameter. This limitation was also apparent with respect to PJT intensity, which was not reported in several studies. Second, even though the included studies did not specify any adverse health events associated with the PJT interventions, it remains unclear whether there was an attempt by the researchers to comprehensively record all possible negative responses. Therefore, to expand our knowledge on the safety of this type of training, future studies should report injuries, pain, or other adverse events related to PJT. Third, we could not compute a meta-analysis for all dynamic and static balance tests (e.g., balance-error score system) due to limited availability of studies reporting these outcomes. Fourth, the moderator effect of subgroups (e.g., age, sex, training background) could not be determined for all balance measures due to limited number of studies. Fifth, most participants in the included studies were relatively young (<30 years of age), and although our moderator analyses indicated no effect of age on PJT related balance outcomes there is a need to study this issue with master athletes and even older adults. In this sense, our results are somewhat limited in their generalizability, and demonstrate that there is a gap on the literature. Finally, we did not include articles written in languages other than English. However, considering that only 0.4% of peer-reviewed PJT studies are written in non-English languages (Ramírez-Campillo et al., 2018), this issue probably had a trivial impact on our findings. Despite these limitations, our systematic review with meta-analysis makes a novel and significant contribution to the existing literature and highlights the benefits of PJT if the goal is to not only improve muscle strength and power but also balance.

### Practical Applications

Findings from this study have practical implications for coaches and practitioners. First, the results of this meta-analysis demonstrate the effectiveness of PJT on measures of dynamic and static balance. Given that balance represents a foundational fitness component for the performance of everyday (e.g., walking on uneven ground) and sport-related activities (e.g., change-of-direction tasks), it should be promoted through balance training and/or PJT (Behm et al., 2010; Gebel et al., 2018). More specifically, our sub-analyses indicate that PJT as positive effects on balance, irrespective of age and sex that are even comparable to those of balance training. However, caution is needed when it comes to the prescription of PJT to avoid overload and subsequent injuries. Third, the implementation of PJT is inexpensive compared to other training methods, requiring little or no equipment, usually involving drills with the body mass used as load (Ramírez-Campillo et al., 2020b). Additionally, PJT may be conducted in a relatively small space, which may be an important advantage during certain scenarios (e.g., encountering pandemic restrictions) where athletes may be forced to train at home (Gentil et al., 2020). Moreover, PJT is a highly variable exercise type compared with other training methods (e.g., flexibility, endurance). This is of particular importance for young athletes (Ward et al., 2007). Fourth, our meta-analysis revealed that other types of training practices such as resistance, balance and whole-body vibration training elucidated similar effects on balance performance compared to PJT. Plyometric exercises appear to induce adaptive processes in the muscle (Moran et al., 2021; Ramírez-Campillo et al., 2022) and the neural system (Ramírez-Campillo et al., 2022) that promote dynamic and static balance performance and kinesthetic control (Lee et al., 2020). Therefore, PJT might not only be a useful tool for increasing muscle power output of the lower limbs, but also balance (Vetrovsky et al., 2019).

## Conclusions

To our knowledge, this is the first meta-analysis to have specifically evaluated the effect of PJT on overall, dynamic and static balance in healthy participants. A total of 1,806 participants, divided into experimental and control groups, were analyzed in our meta-analysis. This large sample size is a strength of the current systematic review and meta-analysis as it addresses the issue of underpowered studies due to smaller sample size, commonly occurring in PJT literature. Our findings demonstrate that compared to active controls, PJT is effective in enhancing various measures of balance performance (dynamic and static), irrespective of the sex and age of the participants. Further, PJT induced similar improvements in balance performance when compared to other training methods (e.g., balance training). Therefore, PJT can also be used as a potential training method for improving balance performance, in conjunction with other physical characteristics such as muscular strength and power. Although our moderator analysis revealed no particular dose-response trend, from 38 studies included in the meta-analysis, the PJT interventions lasted an average of 8 weeks, and the mean weekly frequency of PJT was 2 sessions/week. These programming variables may be considered to improve dynamic and static balance performance by practitioners while designing and implementing PJT programme in the athletic and non-athletic population. Our study further indicated that athletes show greater improvement in balance measures (dynamic and static) compared to non-athletes. Therefore, PJT might be a useful addition to their training regimen to improve balance performance during various dynamic athletic movements. The studies included in our meta-analysis did not report any training related injuries in the recruited participants. Therefore, our systematic review and meta-analysis further confirm the safety and efficacy of PJT in healthy participants of different sex, age and sporting background.

## Data Availability Statement

The original contributions presented in the study are included in the article/Supplementary Material, further inquiries can be directed to the corresponding author/s.

## Author Contributions

All authors listed have made substantial, direct and intellectual contributions to the work, and approved the manuscript for publication.

## Funding

The authors acknowledge the support of the Deutsche Forschungsgemeinschaft and Open Access Publishing Fund of University of Potsdam.

## Conflict of Interest

The authors declare that the research was conducted in the absence of any commercial or financial relationships that could be construed as a potential conflict of interest.

## Publisher's Note

All claims expressed in this article are solely those of the authors and do not necessarily represent those of their affiliated organizations, or those of the publisher, the editors and the reviewers. Any product that may be evaluated in this article, or claim that may be made by its manufacturer, is not guaranteed or endorsed by the publisher.
