# Supplementary material for: Effects of Plyometric Jump Training on Balance Performance in Healthy Participants: A Systematic Review With Meta-Analysis
Source: Front Physiol. 2021 Oct 20;12:730945. doi: 10.3389/fphys.2021.730945 (PMC8564501; doi:10.3389/fphys.2021.730945)
Supplement: Supplementary file 1 [file Table_1.docx]

**APPENDIX**

**SUPPLEMENTARY FILE 1**

**Balance Measures**

*Y-Balance Test:* In order to include studies in the meta-analysis, for those studies that applied more than one measurement from the Y-Balance test in a given group, the following hierarchical criteria was used to select the most representative measurement for balance assessment: i) dominant leg (or right leg for most participants); ii) anterior over posteromedial and posterolateral measures since an inter-limb difference of >4 cm in the anterior direction is associated with a higher risk of lower extremity injury (Plisky et al., 2006; Smith et al., 2015); iii) anterior measures are more sensitive (74.4%) compared to posteromedial (48.8%) and posterolateral directions (59.8%) (Lai et al., 2017); iv) composite scores measured as percentage since a reach distance <94.0% of limb length may predict lower limb injury (e.g., female high school basketball players) (Lai et al., 2017).

*SEBT:* In order to include studies in the meta-analysis, for those studies that applied more than one measurement from the SEBT in a given group, the following hierarchical criteria was used to select the most representative measurement for balance assessment: i) dominant leg (or right leg for most participants), ii) anterior over posteromedial and posterolateral measures since an inter-limb difference of >4 cm in the anterior direction is associated with a higher risk of lower extremity injury (Plisky et al., 2006; Smith et al., 2015), iii) anterior measures are more sensitive (74.4%) compared to posteromedial (48.8%) and posterolateral directions (59.8%) (Lai et al., 2017).

*Laboratory dynamic test*: In order to include studies in the meta-analysis, for those studies that applied more than one measurement from the laboratory dynamic tests in a given group, the following hierarchical criteria was used to select the most representative measurement for balance assessment: i) eyes open, as this is pertinent to athlete (i.e., greater ecological validity) (Springer et al., 2007); ii) anterior over posteromedial and posterolateral measures since an inter-limb difference of >4 cm in the anterior direction is associated with a higher risk of lower extremity injury (Plisky et al., 2006; Smith et al., 2015); iii) path length over other measures (i.e., velocity; surface area) due to better reliability (ICC>0.7) in the former [1]; iv) antero-posterior measures over medio-lateral since the former has better sensitivity for identifying participants with high risk of falling (0.78 vs 0.70) (Kozinc et al., 2020).

*Other dynamic test*: In order to include studies in the meta-analysis, for those studies that applied more than one measurement from the BESS in a given group, single foam stance (r = 0.79) was chosen as it has better agreement as compared to double foam (r = 0.31) and tandem foam stance (r = 0.64) (Bell et al., 2011).

*Standing stork-flamingo test.* In order to include studies data in the meta-analyses, for those studies that applied more than one measurement from the standing stork-flamingo test in a given group, the following hierarchical criteria was used to select the most representative measurement for balance assessment: i) dominant leg (or right leg, for most participants); ii) most reliable measurement (when reported); iii) anterior over posteromedial and posterolateral measures since an inter-limb difference of >4 cm in the anterior direction is associated with a higher risk of lower extremity injury (Plisky et al., 2006; Smith et al., 2015); iv) antero-posterior measures over medio-lateral since the former has better sensitivity for identifying participants with high risk of falling (0.78 vs 0.70) (Kozinc et al., 2020).

*Laboratory static tests*: In order to include studies data in the meta-analyses, for those studies that applied more than one measurement from laboratory static tests in a given group, the following hierarchical criteria was used to select the most representative measurement for balance assessment: i) eyes open, as this is pertinent to athlete (i.e., greater ecological validity) (Springer et al., 2007); ii) anterior over posteromedial and posterolateral measures since an inter-limb difference of >4 cm in the anterior direction is associated with a higher risk of lower extremity injury (Plisky et al., 2006; Smith et al., 2015); iii) antero-posterior measures over medio-lateral since the former has better sensitivity for identifying participants with high risk of falling (0.78 vs 0.70) (Kozinc et al., 2020).

*Other static test*: In order to include studies data in the meta-analyses, for those studies that applied more than one measurement from the Romberg’s test in ad given group, dominant leg (or right leg for most participants) was selected. Moreover, studies that have reported more than one measure for BESS, single firm (r = 0.42) stance was selected as it has better agreement compared the other stances (i.e., double firm; tandem firm) (Bell et al., 2011).

**References:**

Bell, D. R., Guskiewicz, K. M., Clark, M. A., and Padua, D. A. (2011). Systematic review of the balance error scoring system. *Sports Health* 3, 287–295. doi:10.1177/1941738111403122.

Kozinc, Ž., Löfler, S., Hofer, C., Carraro, U., and Šarabon, N. (2020). Diagnostic balance tests for assessing risk of falls and distinguishing older adult fallers and non-fallers: A systematic review with meta-analysis. *Diagnostics* 10, 1–16. doi:10.3390/diagnostics10090667.

Lai, W. C., Wang, D., Chen, J. B., Vail, J., Rugg, C. M., and Hame, S. L. (2017). Lower quarter y-balance test scores and lower extremity injury in NCAA division i athletes. *Orthop. J. Sport. Med.* 5, 1–5. doi:10.1177/2325967117723666.

Nagymáté, G., Orlovits, Z., and Kiss, R. M. (2019). Reliability analysis of a sensitive and independent stabilometry parameter set. *PLoS One* 13, 1–14. doi: 10.1371/journal.pone.0195995.

Plisky, P. J., Rauh, M. J., Kaminski, T. W., and Underwood, F. B. (2006). Star excursion balance test as a predictor of lower extremity injury in high school basketball players. *J. Orthop. Sports Phys. Ther.* 36, 911–919. doi:10.2519/jospt.2006.2244.

Smith, C. A., Chimera, N. J., and Warren, M. (2015). Association of Y balance test reach asymmetry and injury in Division I Athletes. *Med. Sci. Sports Exerc.* 47, 136–141. doi:10.1249/MSS.0000000000000380.

Springer, B. A., Marin, R., Cyhan, T., Roberts, H., and Gill, N. W. (2007). Normative values for the unipedal stance test with eyes open and closed. *J. Geriatr. Phys. Ther.* 30, 8–15. doi:10.1519/00139143-200704000-00003.

**SUPPLEMENTARY FILE 2**

**OVERALL BALANCE**

No significant sub-group difference in overall balance (*p =* 0.719) was found when PJT programmes were applied in males (40 experimental groups; ES = 0.45; 95% CI = 0.27 to 0.63; within-group I^2^ = 52.7%), females (24 experimental groups; ES = 0.53; 95% CI = 0.27 to 0.80; within-group *I^2^* = 57.1%), or mixed samples of males and females (6 experimental groups; ES = 0.24; 95% CI = -0.50 to 0.97; within-group I^2^ = 70.4%).

No significant sub-group difference in overall balance (*p =* 0.150) was found when PJT programmes were applied in participants of ≤15.7 years of age (35 experimental groups; ES = 0.36; 95% CI = 0.16 to 0.55; within-group *I^2^* = 52.5%) or >15.7 years of age (35 experimental groups; ES = 0.57; 95% CI = 0.35 to 0.79; within-group I^2^ = 54.7%).

No significant sub-group difference in overall balance (*p =* 0.219) was found when PJT programmes <8 weeks in duration (52 experimental groups; ES = 0.51; 95% CI = 0.33 to 0.69; within-group *I^2^* = 61.6%) were compared to PJT programmes ≥8 weeks in duration (18 experimental groups; ES = 0.34; 95% CI = 0.14 to 0.54; within-group I^2^ = 12.5%).

No significant sub-group difference in overall balance (*p =* 0.253) was found when PJT programmes with a frequency of ≤2 sessions/week (49 experimental groups; ES = 0.39; 95% CI = 0.24 to 0.55; within-group *I^2^* = 46.3%) were compared to PJT programmes with a frequency of >2 sessions/week (21 experimental groups; ES = 0.60; 95% CI = 0.29 to 0.91; within-group I^2^ = 64.5%).

No significant sub-group difference in overall balance (*p =* 0.092) was found when PJT programmes with ≤18 total sessions (43 experimental groups; ES = 0.36; 95% CI = 0.18 to 0.55; within-group *I^2^* = 52.8%) were compared to PJT programmes with >18 total sessions (27 experimental groups; ES = 0.61; 95% CI = 0.39 to 0.83; within-group I^2^ = 52.8%).

**OVERALL DYNAMIC BALANCE**

No significant sub-group difference in overall dynamic balance (*p =* 0.712) was found when PJT programmes were applied in males (21 experimental groups; ES = 0.55; 95% CI = 0.34 to 0.76; within-group I^2^ = 16.6%) or females (14 experimental groups; ES = 0.53; 95% CI = 0.18 to 0.89; within-group *I^2^* = 68.6%).

No significant sub-group difference in overall dynamic balance (*p =* 0.498) was found when PJT programmes were applied in participants of ≤15.9 years of age (19 experimental groups; ES = 0.57; 95% CI = 0.30 to 0.84; within-group *I^2^* = 58.0%) or >15.9 years of age (18 experimental groups; ES = 0.43; 95% CI = 0.10 to 0.75; within-group I^2^ = 56.0%).

No significant sub-group difference in overall dynamic balance (*p =* 0.552) was found when PJT programmes <8 weeks in duration (17 experimental groups; ES = 0.57; 95% CI = 0.17 to 0.97; within-group *I^2^* = 71.4%) were compared to PJT programmes ≥8 weeks in duration (20 experimental groups; ES = 0.44; 95% CI = 0.24 to 0.64; within-group I^2^ = 24.1%).

No significant sub-group difference in overall dynamic balance (*p =* 0.737) was found when PJT programmes with a frequency of ≤2 sessions/week (25 experimental groups; ES = 0.54; 95% CI = 0.31 to 0.76; within-group *I^2^* = 51.6%) were compared to PJT programmes with a frequency of >2 sessions/week (12 experimental groups; ES = 0.44; 95% CI = -0.04 to 0.93; within-group I^2^ = 66.7%).

No significant sub-group difference in overall dynamic balance (*p =* 0.400) was found when PJT programmes with ≤18 total sessions (24 experimental groups; ES = 0.54; 95% CI = 0.27 to 0.82; within-group *I^2^* = 63.0%) were compared to PJT programmes with >18 total sessions (13 experimental groups; ES = 0.38; 95% CI = 0.10 to 0.66; within-group I^2^ = 31.0%).

**Y-BALANCE TEST**

No significant sub-group difference in dynamic balance measured through the YBT (*p =* 0.697) was found when PJT programmes were applied in participants of ≤16.3 years of age (9 experimental groups; ES = 0.62; 95% CI = 0.18 to 1.07; within-group *I^2^* = 62.8%) or >16.3 years of age (8 experimental groups; ES = 0.50; 95% CI = 0.06 to 0.94; within-group I^2^ = 52.8%).

No significant sub-group difference in dynamic balance measured through the YBT (*p =* 0.347) was found when PJT programmes ≤8 weeks in duration (12 experimental groups; ES = 0.66; 95% CI = 0.28 to 1.05; within-group *I^2^* = 57.3%) were compared to PJT programmes >8 weeks in duration (5 experimental groups; ES = 0.35; 95% CI = -0.17 to 0.87; within-group I^2^ = 57.2%).

No significant sub-group difference in dynamic balance measured through the YBT (*p =* 0.170) was found when PJT programmes with a frequency of ≤2 sessions/week (13 experimental groups; ES = 0.43; 95% CI = 0.14 to 0.73; within-group *I^2^* = 45.5%) were compared to PJT programmes with a frequency of >2 sessions/week (4 experimental groups; ES = 1.13; 95% CI = 0.18 to 2.07; within-group I^2^ = 69.4%).

No significant sub-group difference in dynamic balance measured through the YBT (*p =* 0.946) was found when PJT programmes with ≤18 total sessions (11 experimental groups; ES = 0.57; 95% CI = 0.23 to 0.92; within-group *I^2^* = 50.6%) were compared to PJT programmes with >18 total sessions (6 experimental groups; ES = 0.60; 95% CI = -0.05 to 1.24; within-group I^2^ = 67.8%).

**STAR EXCURSION BALANCE TEST**

Moderator analyses according to participant’s sex and total number of PJT sessions was precluded as less than three studies provided dynamic balance data, measured through the SEBT, for the aforementioned moderators.

No significant sub-group difference in dynamic balance measured through the SEBT (*p =* 0.130) was found when PJT programmes were applied in participants of ≤15.6 years of age (5 experimental groups; ES = 0.78; 95% CI = 0.37 to 1.20; within-group *I^2^* = 30.7%) or >15.6 years of age (4 experimental groups; ES = 0.02; 95% CI = -0.88 to 0.92; within-group I^2^ = 76.6%).

No significant sub-group difference in dynamic balance measured through the SEBT (*p =* 0.495) was found when PJT programmes ≤6 weeks in duration (5 experimental groups; ES = 0.28; 95% CI = -0.59 to 1.14; within-group *I^2^* = 83.9%) were compared to PJT programmes >6 weeks in duration (4 experimental groups; ES = 0.61; 95% CI = 0.18 to 1.04; within-group I^2^ = 0.0%).

Significant sub-group difference in dynamic balance measured through the SEBT (*p =* 0.044) was found when PJT programmes with a frequency of ≤2 sessions/week (4 experimental groups; ES = 0.89; 95% CI = 0.45 to 1.33; within-group *I^2^* = 30.3%) were compared to PJT programmes with a frequency of >2 sessions/week (5 experimental groups; ES = 0.05; 95% CI = -0.65 to 0.74; within-group I^2^ = 67.1%).

**LABORATORY-BASED DYNAMIC TESTS**

No significant sub-group difference in dynamic balance measured through laboratory-based equipment (*p =* 0.606) was found when PJT programmes were applied in participants of ≤15.9 years of age (5 experimental groups; ES = 0.21; 95% CI = -0.20 to 0.62; within-group *I^2^* = 0.0%) or >15.9 years of age (4 experimental groups; ES = 0.38; 95% CI = -0.09 to 0.84; within-group I^2^ = 0.0%).

No significant sub-group difference in dynamic balance measured through laboratory-based equipment (*p =* 0.565) was found when PJT programmes ≤6 weeks in duration (5 experimental groups; ES = 0.39; 95% CI = -0.09 to 0.87; within-group *I^2^* = 0.0%) were compared to PJT programmes >6 weeks in duration (4 experimental groups; ES = 0.21; 95% CI = -0.20 to 0.61; within-group I^2^ = 0.0%).

No significant sub-group difference in dynamic balance measured through laboratory-based equipment (*p =* 0.944) was found when PJT programmes with a frequency of ≤2 sessions/week (6 experimental groups; ES = 0.29; 95% CI = -0.07 to 0.65; within-group *I^2^* = 0.0%) were compared to PJT programmes with a frequency of >2 sessions/week (3 experimental groups; ES = 0.26; 95% CI = -0.34 to 0.86; within-group I^2^ = 0.8%).

No significant sub-group difference in dynamic balance measured through laboratory-based equipment (*p =* 0.530) was found when PJT programmes with ≤18 total sessions (6 experimental groups; ES = 0.37; 95% CI = -0.04 to 0.77; within-group *I^2^* = 0.0%) were compared to PJT programmes with >18 total sessions (3 experimental groups; ES = 0.17; 95% CI = -0.31 to 0.64; within-group I^2^ = 0.0%).

**OVERALL STATIC BALANCE**

No significant sub-group difference in overall static balance (*p =* 0.712) was found when PJT programmes were applied in males (19 experimental groups; ES = 0.56; 95% CI = 0.26 to 0.87; within-group I^2^ = 52.0%), females (10 experimental groups; ES = 0.41; 95% CI = 0.18 to 0.63; within-group *I^2^* = 9.3%) or mixed samples of males and females (4 experimental groups; ES = 0.50; 95% CI = 0.09 to 0.91; within-group I^2^ = 0.5%).

No significant sub-group difference in overall static balance (*p =* 0.543) was found when PJT programmes were applied in participants of ≤14.6 years of age (17 experimental groups; ES = 0.43; 95% CI = 0.24 to 0.62; within-group *I^2^* = 0.0%) or >14.6 years of age (16 experimental groups; ES = 0.55; 95% CI = 0.23 to 0.86; within-group I^2^ = 57.4%).

No significant sub-group difference in overall static balance (*p =* 0.821) was found when PJT programmes <8 weeks in duration (15 experimental groups; ES = 0.51; 95% CI = 0.20 to 0.83; within-group *I^2^* = 47.6%) were compared to PJT programmes ≥8 weeks in duration (18 experimental groups; ES = 0.47; 95% CI = 0.26 to 0.68; within-group I^2^ = 29.4%).

No significant sub-group difference in overall static balance (*p =* 0.202) was found when PJT programmes with a frequency of ≤2 sessions/week (23 experimental groups; ES = 0.41; 95% CI = 0.25 to 0.57; within-group *I^2^* = 2.1%) were compared to PJT programmes with a frequency of >2 sessions/week (10 experimental groups; ES = 0.74; 95% CI = 0.26 to 1.22; within-group I^2^ = 66.7%).

No significant sub-group difference in overall static balance (*p =* 0.689) was found when PJT programmes with ≤18 total sessions (19 experimental groups; ES = 0.53; 95% CI = 0.35 to 0.71; within-group *I^2^* = 0.0%) were compared to PJT programmes with >18 total sessions (14 experimental groups; ES = 0.45; 95% CI = 0.11 to 0.80; within-group I^2^ = 59.3%).

**FLAMINGO/STORK TESTS**

No significant sub-group difference in static balance measured through the flamingo and stork tests (*p =* 0.314) was found when PJT programmes were applied in males (12 experimental groups; ES = 0.61; 95% CI = 0.17 to 1.05; within-group I^2^ = 69.1%) or females (4 experimental groups; ES = 0.20; 95% CI = -0.47 to 0.87; within-group *I^2^* = 72.9%) or

No significant sub-group difference in static balance measured through the flamingo and stork tests (*p =* 0.459) was found when PJT programmes were applied in participants of <14.5 years of age (9 experimental groups; ES = 0.35; 95% CI = 0.00 to 0.70; within-group *I^2^* = 50.1%) or ≥14.5 years of age (8 experimental groups; ES = 0.65; 95% CI = -0.07 to 1.37; within-group I^2^ = 80.6%).

No significant sub-group difference in static balance measured through the flamingo and stork tests (*p =* 0.334) was found when PJT programmes <8 weeks in duration (4 experimental groups; ES = 1.03; 95% CI = -0.27 to 2.32; within-group *I^2^* = 88.1%) were compared to PJT programmes ≥8 weeks in duration (13 experimental groups; ES = 0.37; 95% CI = 0.04 to 0.70; within-group I^2^ = 55.8%).

No significant sub-group difference in static balance measured through the flamingo and stork tests (*p =* 0.130) was found when PJT programmes with a frequency of ≤2 sessions/week (13 experimental groups; ES = 0.28; 95% CI = -0.02 to 0.59; within-group *I^2^* = 55.4%) were compared to PJT programmes with a frequency of >2 sessions/week (4 experimental groups; ES = 1.48; 95% CI = -0.04 to 3.01; within-group I^2^ = 85.9%).

No significant sub-group difference in static balance measured through the flamingo and stork tests (*p =* 0.931) was found when PJT programmes with <20 total sessions (7 experimental groups; ES = 0.44; 95% CI = -0.06 to 0.94; within-group *I^2^* = 70.9%) were compared to PJT programmes with ≥20 total sessions (10 experimental groups; ES = 0.47; 95% CI = -0.05 to 0.99; within-group I^2^ = 71.5%).

**LABORATORY-BASED STATIC TESTS**

No significant sub-group difference in static balance measured through laboratory-based equipment (*p =* 0.744) was found when PJT programmes were applied in males (7 experimental groups; ES = 0.55; 95% CI = 0.15 to 0.94; within-group I^2^ = 0.0%), females (4 experimental groups; ES = 0.36; 95% CI = -0.03 to 0.76; within-group *I^2^* = 17.7%), or mixed samples of males and females (3 experimental groups; ES = 0.59; 95% CI = 0.03 to 1.14; within-group I^2^ = 23.4%).

No significant sub-group difference in static balance measured through laboratory-based equipment (*p =* 0.272) was found when PJT programmes were applied in participants of <18 years of age (10 experimental groups; ES = 0.39; 95% CI = 0.11 to 0.67; within-group *I^2^* = 0.0%) or ≥18 years of age (4 experimental groups; ES = 0.67; 95% CI = 0.25 to 1.09; within-group I^2^ = 0.0%).

No significant sub-group difference in static balance measured through laboratory-based equipment (*p =* 0.925) was found when PJT programmes ≤6 weeks in duration (9 experimental groups; ES = 0.49; 95% CI = 0.17 to 0.82; within-group *I^2^* = 0.0%) were compared to PJT programmes >6 weeks in duration (5 experimental groups; ES = 0.47; 95% CI = 0.08 to 0.86; within-group I^2^ = 28.2%).

No significant sub-group difference in static balance measured through laboratory-based equipment (*p =* 0.504) was found when PJT programmes with a frequency of ≤2 sessions/week (9 experimental groups; ES = 0.40; 95% CI = 0.08 to 0.72; within-group *I^2^* = 0.0%) were compared to PJT programmes with a frequency of >2 sessions/week (5 experimental groups; ES = 0.56; 95% CI = 0.21 to 0.91; within-group I^2^ = 9.0%).

No significant sub-group difference in static balance measured through laboratory-based equipment (*p =* 0.540) was found when PJT programmes with ≤13 total sessions (7 experimental groups; ES = 0.57; 95% CI = 0.18 to 0.97; within-group *I^2^* = 0.0%) were compared to PJT programmes with >13 total sessions (7 experimental groups; ES = 0.42; 95% CI = 0.14 to 0.71; within-group I^2^ = 0.0%).

**Supplementary Table 1.**

| **Study** | **Balance test** | **Measurement and assessment procedure** |
| --- | --- | --- |
| Akin and Kesilmis, 2020 | Laboratory-based dynamic balance test | Bipedal balance measurements were conducted on a Prokin Tecno Body equipment (PKW 200 PL, Italy) with mono-axial base for antero-posterior sway. Perimeter-length measurements started when the participant adopted the balance position with barefoot and thin sportswear. Measurements were taken twice after 30 s and 60 s rest intervals. |
| Alikhani et al., 2019 | Y-balance test | The test was conducted using a Y balance test device. The device consists of a stance platform from which three pieces of polyvinylchloride pipe project in the anterior, posteromedial, and posterolateral reach directions. The participant stood on a center footplate to perform the test. While maintaining single-limb stance on the dominant limb, the participant reached with the other limb in the anterior, posteromedial, and posterolateral directions in relation to the stance foot by pushing a reach indicator as far as possible. The participant pushed the reach indicator along the pipe with their dominant limb, and the reach indicator remains over the tape measure after performance of the test to allow for easy measurement |
| Arabatzi, 2018 | Laboratory-based static balance test | Participants were instructed to stand erect, as motionless as possible, on a normal comfortable posture, with opened eyes looking straight ahead at a cross marked at ap­proximately eye level on a black board 3 m away and barefoot with feet shoulder width apart on the platform with the arms by their sides. Each participant was requested to keep a quiet stance posture for 30 seconds. The as­sessment included 3 measurements, and 5-min rest was provided between successive trials. The best trial was further analyzed. Displacements of center of pressure were performed on a Kistler piezoelectric force platform. Centre of pressure (CoP) in anterior/posterior (A/P) and medio/lateral (M/L) CoP displacement (mm) was determined from ground reaction forces. |
|  | Laboratory-based static balance test | The participants were instructed to stand on one foot, which was placed pointing straight forward in relation to reference lines in the frontal and sagit­tal planes. The swinging leg was flexed 90° at the hip and knee joints with both arms hanging relaxed at the sides. The subjects were instructed to stand as still as possible, looking straight ahead at a point on the wall 65 cm away. The test order between legs was randomized. Data recording started once the subject was stable in the required posture. Ample time was provided for famil­iarization. If one-leg balance was not maintained for 10 seconds the trial was not recorded and the measurement was repeated. Displacements of center of pressure were performed on a Kistler piezoelectric force platform. Centre of pressure (CoP) in anterior/posterior and medio/lateral CoP displacement (mm) was determined from ground reaction forces. |
| Asadi et al., 2015 | SEBT | The SEBT procedures involved reaching in the anterolateral, anterior, anteromedial, medial, posteromedial, posterior, posterolateral, and lateral reach directions. While maintaining a single-leg stance, each participant was asked to maximally reach along the identified line with the contralateral limb and lightly touch the line with the distal part of the foot. All reach trials began with both feet in contact with the ground and with the stance leg appropriately positioned in relation to the center of the SEBT grid. The participants were instructed to keep their hands on their hips while performing this task. Failed test criteria included the following: (a) if the reach foot was placed in contact with the ground for support, (b) if the stance foot was moved or lifted, (c) if equilibrium was lost during any part of the reach or return phase, and (d) if the point of contact was to either side of the taped line. A total of 5 test reaches were performed in each direction. Reach distance was quantified by measuring the distance (in centimeters) from the center of the crosshairs to the point of distal foot-ground contact marked in ink by the investigator. Performance of SEBT for each direction was represented by an average of the 3 best reach distances and normalized to leg length. A 1-minute rest was allowed between directions. The order of the reach direction was counterbalanced for all participants. Participant’s legs were measured from the anterior superior iliac spine to the distal tip of the medial malleolus using a standard tape measure while participants lay supine. Leg length was used to normalize excursion distances by dividing the distance reached by leg length then multiplying by 100. |
| Benis et al., 2016 | Y-balance test | The test was conducted using a Y balance test device comprising a stance platform to which three pieces of polyvinyl chloride pipe were attached in the anterior, posteromedial, and posterolateral reach directions. The posterior pipes were positioned 135 degree from the anterior pipe with 45 degree between them. Each pipe was marked in 5-mm increments. The test was performed with the distal aspect of the great toe centered at the junction of the Y balance test device. The participant had to reach with the opposite leg in the anterior, posteromedial, and posterolateral directions and push a target (reach indicator) along the pipe that standardized the reach distance; the target remained over the tape measure after completion of the test. Reach distance was measured from the most distal aspect of the toes of the stance foot to the most distal aspect of the reach foot in the anterior, posteromedial, and posterolateral directions. The test scores were analyzed using the average of three trials for each reach direction for each lower extremity, as well as the average of the total of the reach directions (composite score). The test composite score was calculated by dividing the sum of the maximum reach distance in the anterior, posteromedial, and posterolateral directions by three times the limb length of the participant and then multiplying by 100: ([anterior + posteromedial + posterolateral]/[leg length×3])×100. The ICC (1,1) of anterior, posteromedial, posterolateral and composite Y-balance test scores were 0.91, 0.88, 0.83, and 0.90, respectively, for the right limb and 0.88, 0.90, 0.82, and 0.89, respectively, for the left limb. |
| Bouteraa et al., 2020 | Stork test | The test was performed on the dominant leg. Subjects stood with their opposite foot resting against the inside of the supporting knee and both hands on the hips. On the “go” signal, they raised the heel of their foot from the floor. The posture was held as long as possible, but the test was terminated when the heel of the supporting leg touched the ground or the foot moved away from the knee cap. The ICC was 0.90. |
|  | Y-balance test | Dynamic balance was assessed for the dominant leg. Barefoot subjects maintain a single leg stance while reaching as far as possible with the contralateral leg in three directions (anterior, posteromedial and posterolateral) set by fixing tape measures to the floor. The two posterior lines extended at an angle of 135° from the anterior line. Athletes’ leg lengths were first determined while lying supine, measuring from the anterior superior iliac spine to the most distal aspect of the medial malleolus. They then stood with the tip of their great toe at the center of the grid. They were asked to reach in the three directions while maintaining a single-limb stance. The maximal reach was measured in each direction. The composite score was calculated as: ([maximum anterior reach distance + maximum posteromedial reach distance + maximum posterolateral reach distance] / [leg length × 3] × 100). Test-retest reliability for the different reach directions ranged between 0.90 and 0.95. |
| Chaouachi et al., 2014b | Stork test | The participants stood with their opposite foot against the inside of the supporting knee, and both hands on his hips. On the command, the subject raised the heel of their foot from the floor and attempted to maintain their balance as long as possible. The trial ended if the subject moved his hands from his hips, the ball of the dominant foot moved from its original position, or if the heel touched the floor. This test was carried out on the dominant leg acting as the standing leg. The test was timed (in seconds) using a stopwatch. The total time was recorded in seconds. The score was the best of three attempts. |
|  | SEBT | The test consisted of eight lines of cloth measuring tape adhered to the floor with clear packing tape 45° apart from each other, in the shape of an asterisk (star). While testing, subjects stood on one leg in the middle of the star and reached as far as possible along each of the 8 directions with their toes of the opposite leg, holding their hands on their hips, and keeping the heel of the stance leg on the ground. Each distance was read from the center of the star to the mark. Distances were measured in centimetres and normalized by dividing by the subject’s lower extremity length (anterior-superior iliac spine to distal end of the medial malleolus) and multiplying by 100. |
| Cherni et al., 2019 | Laboratory-based static balance test | Balance Postural control was assessed using a 3strain gauge force platform (PostureWin©, Techno Concept, Mane, France) with a sampling frequency of 40Hz and 12 bits analog/digital conversion. Balance was tested with the eyes both open and closed, under static conditions (stable ground). Instability was measured in the antero-posterior and medio-lateral directions. Subjects stood barefoot, with their arms by their sides. With their eyes open, they looked at a fixed-level target (1 cm2) set at a distance of 2 m, and with their eyes closed, they maintained the same posture. The six postural conditions were assessed in random order. The test lasted 51.2 s and in each dynamic condition, the subject maintained the seesaw platform as horizontal as possible for 25.6 s. A total loss of balance invalidated the trial. |
|  | Laboratory-based dynamic balance test | Postural control was assessed using a 3-strain gauge force platform (PostureWin©, Techno Concept, Mane, France) with a sampling frequency of 40Hz and 12 bits analog/digital conversion. Balance was tested with the eyes both open and closed, under dynamic conditions (lying on a see-saw device with a 55 cm cylinder radius and a height of 6 cm). |
| Cigerci and Genc, 2020 | SEBT | The participants were asked to touch the furthest point they could reach in eight directions with angle of 45 degrees. The test was performed three times for both feet. Balance score was calculated by the sum of the eight directions/leg length*100. |
| Drouzas et al., 2020 | Flamingo test | The participants were asked to stand on one leg for one minute, while the other leg was kept flexed at the knee joint and griped with the same side arm with the foot close to the buttocks. The final score (lower scores indicate better balance) was calculated as the average of the right and left leg performance, which was defined as the number of times the participant lost their balance. |
| Hammami et al., 2019c | Stork test | Participants stood with one foot positioned against the inside of the supporting knee and both hands on their hips. On command, they raised their heel from the floor and maintained their balance as long as possible. The trial ended if the subject moved her hands from the hips, the ball of the support leg moved from its original position, or if the heel touched the floor. Tests were carried out standing on the right and left legs, with the eyes open. |
|  | Y-balance test | Reach directions were evaluated by affixing tape measures to the floor, one oriented anterior to the apex and two others aligned at 135° in the posterior-medial and posterior-lateral directions. Subjects stood on the dominant leg, with the most distal aspect of their great toe at the centre of the grid. They then reached in the specified direction, while maintaining a single-limb stance. A test was classified as invalid if the participants (1) did not touch the line with the reach foot while maintaining weight bearing on the stance leg, (2) lifted the stance foot from the center grid, (3) lost balance at any point during the trial, (4) did not maintain start and return positions for a full second, or (5) touched down the reach foot to gain considerable support. Variables included the maximal reach in each direction and the average maximum normalized reach across the three directions. Measures were normalized by dividing the excursion distance by the participant’s leg length, then multiplying by 100. |
| Hammami et al., 2019a | Stork test | Participants stood with one foot positioned against the inside of the supporting knee and both hands on their hips. On command, they raised their heel from the floor and maintained their balance as long as possible. The trial ended if the subject moved her hands from the hips, the ball of the support leg moved from its original position, or if the heel touched the floor. Tests were carried out standing on the right and left legs, with the eyes open. Test-retest reliability scores was reported to be high (ICC = 0.812 and ICC = 0.847 for the right and left legs, respectively). |
|  | Y-balance test | Reach directions were evaluated by affixing tape measures to the floor, one oriented anterior to the apex and two others aligned at 135° in the posterior-medial and posterior-lateral directions. Subjects stood on the dominant leg, with the most distal aspect of their great toe at the centre of the grid. They then reached in the specified direction, while maintaining a single-limb stance. A test was classified as invalid if the participants (1) did not touch the line with the reach foot while maintaining weight bearing on the stance leg, (2) lifted the stance foot from the center grid, (3) lost balance at any point during the trial, (4) did not maintain start and return positions for a full second, or (5) touched down the reach foot to gain considerable support. The maximal reach was measured in each direction. Three test trials were conducted in each direction, with 2-minute rest intervals. Test-retest reliability for the different reach directions ranged between 0.90 and 0.95. |
| Hammami et al., 2019b | Stork test | The test was performed in the standard manner, with participants standing on their dominant leg and resting their opposite foot against the inside of the supporting knee. Test - retest reliability scores for such measures was reported to be high (ICC = 0.09) with coefficients of variation of 65.3% and 68.8 % for right and left legs, respectively. |
|  | Y-balance test | Reach directions were evaluated by affixing tape measures to the floor, one oriented anterior to the apex and two others aligned at 135° in the posterior-medial and posterior-lateral directions. Subjects stood on the dominant leg, with the most distal aspect of their great toe at the centre of the grid. They then reached in the specified direction, while maintaining a single-limb stance. A test was classified as invalid if the participants (1) did not touch the line with the reach foot while maintaining weight bearing on the stance leg, (2) lifted the stance foot from the center grid, (3) lost balance at any point during the trial, (4) did not maintain start and return positions for a full second, or (5) touched down the reach foot to gain considerable support. The maximal reach was measured in each direction. Three test trials were conducted in each direction, with 2-minute rest intervals. Test-retest reliabilities for the 3 reach directions were 0.90-0.95, with respective coefficients of variation of 12.7%, 12.0% and 28.5% for the right leg and 12.0%, 12.1% and 25.9% for the left leg. |
| Hammami et al., 2020a | Stork test | The test was performed in the standard manner, with participants standing on their dominant leg and resting their opposite foot against the inside of the supporting knee. Test -retest reliability scores for measurements on the right leg and left legs were 0.784 and 0.773, with respective 95% confidence interval of 0.409–0.847 and 0.617–0.819. |
|  | Y-balance test | Dynamic balance was assessed on the dominant leg, using the Y-balance test. Three trials were conducted in each direction, with two-minute rest intervals. Test-retest reliabilities for the 3 reach directions ranged from 0.869 to 0.911, with respective 95% confidence intervals of 0.783–0.916, 0.814–0.898, 0.784–0.921 for the left, back and right side respectively (right support leg); and 0.845–0.956, 0.874–0.926, 0.805–0.911 for the left, back and right side respectively (left support leg). |
| Hammami et al., 2020c | Stork test | Subjects stood on their dominant leg with their opposite foot resting against the inside of the supporting knee and both hands on their hips. On signal, they raised their heel; the test was terminated when the heel touched the ground or the foot moved away from the patella. Stork right ICC: 0.853; Stork left ICC: 0.637 |
|  | Y-balance test | Dynamic balance was assessed for both right and left legs. Supine leg lengths were first determined from the anterior superior iliac spine to the most distal aspect of the medial malleolus. Subjects then stood barefoot and single-legged, with the tip of their great toe at the centre of the grid, and reached in anterior, posteromedial and posterolateral directions, marked on the floor by tape. The posterior lines extended at an angle of 135° from the anterior line. The maximal reach was measured in each direction, and a composite score calculated as [(maximum anterior + maximum posteromedial + maximum posterolateral reach distance) / (leg length × 3) × 100]. ICC values ranged from 0.894 to 0.977. |
| Hammami et al., 2020b | Stork test | On command, the subject raised the heel of one foot from the floor and placed it against the inside of the supporting knee, with both hands on the hips, maintaining balance for as long as possible. The trial ended if the participant moved his hands from his hips, if the ball of the dominant foot moved from its original position, or if the heel touched the floor. This test was carried out on the dominant leg, with the eyes open. Previous test–retest reliability scores with a similar adolescent population have been high (error of measurement 0.3 to  3.2%). |
|  | Y-balance test | Reach directions were evaluated by affixing tape measures to the floor, one oriented anteriorly, and the other two running at 135° in the posteromedial and posterolateral directions. All testing was conducted barefoot. Subjects stood on the dominant leg, with the most distal aspect of their great toe at the centre of the grid. They then reached in the specified direction, while maintaining a single-limb stance. The average maximum reach across the three directions (normalized for leg length) was calculated as a composite score for each subject. |
| Hopper et al., 2017 | SEBT | The test assessed anterior, posteromedial and posterolateral balance. Each participant completed 6 repetitions of SEBT in all directions (anterior, posteromedial and posterolateral). Analysis of SEBT scores showed excellent between-trial reliability across all directions (ICCa: 0.93). |
| Huang et al., 2014 | Laboratory-based static balance test | Each participant was instructed to perform a single-legged stance on either the dominant side or the affected side with eyes open and eyes closed. Each participant was barefoot and kept the arms crossed at the chest. During the eyes-open condition, he or she was asked to look at an eye-level target 3 m ahead. Each successful trial required the person to remain as motionless as possible for 20 seconds. The standard deviation of the medial-lateral and anterior-posterior Center of Pressure displacement in each trial represented the distribution of the Center of Pressure sway level. The maximum ranges of the medial-lateral and anterior-posterior Center of Pressure were the difference between maximum and minimum in the corresponding axis. The long and short axes of the ellipse were defined by 2 SDs of the medial-lateral and the anterior-posterior Centre of Pressure sway, respectively, and were used to construct the 95% elliptical sway area of Centre of Pressure. |
|  | Laboratory-based static balance test | For the single-legged drop landing, the participant was instructed to hop medially or laterally off a 16-cm-high platform. He or she had to maintain the single-legged stance with hands on the waist while standing on the platform for 3 seconds as a preparatory posture. In response to an auditory cue, the person hopped down onto a force plate (model AM FP4060-07-1000; Bertec Corporation, Columbus, OH) and regained stability as rapidly as possible, keeping the body erect and facing forward. The participant maintained the single legged stance for 5 more seconds. A failure was defined as repetitive hopping on the force plate, taking the foot off the ground to regain balance, or moving the hands away from the waist. The TTS represented the time (sec) taken from foot contact on the ground to the midpoint of the stable condition was computed. |
| Jlid et al., 2019 | SEBT | The test integrated a single-leg stance with maximum reach of the opposite leg. Participants stood in the centre of a grid, with eight lines radiating at 45° increments from the centre of the grid: anterolateral, anterior, anteromedial, medial, posteromedial, posterior, posterolateral, and lateral. Reach distances were normalized by dividing each excursion distance by the participant’s leg length and multiplying the value obtained by 100. ICCs for 3 repeated trials before and after intervention period of the 8 directions using dominant and nondominant legs ranged from 0.90 to 0.94, with 95% CI ranging between 0.88 and 0.97, respectively |
| Jlid et al., 2020 | SEBT | The test was performed with participants standing in the middle of a grid placed on the floor with eight lines extending at 45° increments from the centre of the grid. The eight lines on the grid were named in relation to the direction of reach relative to the stance leg: anterolateral, anterior, anteromedial, medial, posteromedial, posterior, posterolateral, and lateral. The reach distances were normalized by dividing each excursion distance (in cm) by the participant’s leg length (in cm) and then multiplying the value obtained by 100. The ICCs for three trials before and after intervention period of the eight directions using dominant and non-dominant legs ranged from 0.90 to 0.94 (95% CI = 0.88–0.97) |
| Karadenizli, 2016 | Laboratory-based static balance test | The participant stood on the balance platform barefoot, and on one leg (left, and right). The participant was asked to look at the screen in front of him, whit arms at his sides, as motionless as possible, for 30 seconds, without minimal verbal feedback. The data obtained was evaluated in terms of left-foot ellipse area and right-foot ellipse area. |
|  | Laboratory-based dynamic balance test | The test was used as a monoaxial dynamic-time test Mediolateral to one axis at a time, and to assess the participant’s skill required to completing the exercise. In this test, the participant tries to see some balls-objectives that they come against. The subject’s scope was to hit objectives and follow ideal line within 60 second duration (a) hold with two hand and (b) without hold. Subject load was selected 5 hard degree (according to soft (0) to hard (10) degree system |
| Kim and Park, 2016 | Other static balance test | Each subject was instructed to stand with the hip joint and knee joint flexed to 90° with eyes closed and both hands placed on the waist. The measurement was conducted three times repeatedly. The average value was used as the measured value (seconds). |
| Lee et al., 2020 | Laboratory-based dynamic balance test | In a standing position, the subject held his or her pelvis, maintained balance on a single leg, and then reached with the other leg as much as possible in three directions (anterior, posterior–lateral, posterior–medial). The side affected by functional ankle instability was set as the weight-bearing axis. To compensate for leg length differences, normalized composite scores (%) were used, determined by summing the reach distances in each direction, dividing the result by three times the leg length and then multiplying by 100. |
| Lovechhio et al., 2019 | Stork test | Subjects were asked to stand barefoot on their dominant lower limb with the other one raised and parallel to the ground, for as long as possible (i.e., seconds). The hands were on hips and the eyes closed. The trials ended when the subject either: opened eyes, moved his hand/arms, pivoted foot on the ground, moved the raised foot toward or away from the standing leg or touched the floor. (Test–retest reliability coefficient of 0.994 were reported by previous studies |
| Makhlouf et al., 2018 | Stork test | The participants stood with their opposite foot against the inside of the supporting knee and both hands on the hips. On the command, participants raised the heel of their foot from the floor and attempted to maintain their balance as long as possible. The trial ended either if the participant moved his hands from his hips, the ball of the dominant foot moved from its original position, or if the heel touched the floor. This test was carried out on the dominant leg acting as the standing leg. The test was timed (s) using a stopwatch. The recorded score (duration in seconds) was the best of three attempts. |
|  | Y-balance test | Dynamic balance was tested using the lower quarter Y balance test. Before the test started, participants’ length of the right leg was assessed while in a supine lying position by measuring the distance from the anterior superior iliac spine to the most distal aspect of the medial malleolus. Further, participants practiced six trials per reach direction to be familiarized with the testing procedures. All trials were conducted barefooted. Participants stood on the dominant leg, with the most distal aspect of their great toe on the centre of the footplate from the Y balance test kit. The participants were then asked to push the reach-indicator block with the free limb in the anterior, posteromedial, and posterolateral directions in relation to the stance foot on the central footplate, while maintaining their single-limb stance. The average maximum normalized reach across the three directions was calculated in order to record a composite score for each subject. Test measures were normalized by dividing each excursion distance by the participant’s leg length, then multiplying by 100. The examiner manually measured the distance from the centre of the grid to the touch point and the results were documented after each reach. A composite score was calculated and taken as the dependent variable using the following formula: [(maximum anterior reach distance × maximum posteromedial reach distance × maximum posterolateral reach distance) / (leg length × 3)] × 100. |
| McLeod et al., 2009 | Other dynamic balance test | Using three different stances (double-leg, single-leg, and tandem stance), the participants completed six separate 20-second tests on a firm surface. The 6 conditions were randomized. Subjects were instructed to stay in the required stance with their hands on their iliac crests and eyes closed. Once an athlete closed her eyes, the test was started. During the single-leg stances, the subjects stood on their non-dominant limb, as defined as their stance leg during kicking, and were asked to maintain the contralateral limb in 20° of hip flexion and 40° of knee flexion. The non-dominant limb was chosen as the standardized test limb across subjects. Subjects were asked that on losing their balance, they should open their eyes and return to the starting position as quickly as possible. Individual condition scores were calculated by adding the error points in each of the 6 conditions. Trials were considered incomplete if the subject could not remain in the stance position for longer than 5 seconds, in which case she was assigned a standard maximum score of 10 for that stance Pilot studies on BESS scoring demonstrated excellent intratester (ICC = .90) and intertester (ICC = .85) reliability. |
|  | Other dynamic balance test | Using three different stances (double-leg, single-leg, and tandem stance) the participants completed six separate 20-second tests on a foam surface. The 6 conditions were randomized. Subjects were instructed to stay in the required stance with their hands on their iliac crests and eyes closed. Once an athlete closed her eyes, the test was started. During the single-leg stances, the subjects stood on their non-dominant limb, as defined as their stance leg during kicking, and were asked to maintain the contralateral limb in 20° of hip flexion and 40° of knee flexion. The non-dominant limb was chosen as the standardized test limb across subjects. Subjects were asked that on losing their balance, they should open their eyes and return to the starting position as quickly as possible. Individual condition scores were calculated by adding the error points in each of the 6 conditions. Trials were considered incomplete if the subject could not remain in the stance position for longer than 5 seconds, in which case she was assigned a standard maximum score of 10 for that stance Pilot studies on BESS scoring demonstrated excellent intratester (ICC = .90) and intertester (ICC = .85) reliability. |
|  | SEBT | The test setup consisted of five lines at 45° and 90° intervals extending from a centre point. The angles used extended out from the centre at 45°, 90°, 180°, 270°, and 315° to provide targeting for the participants. Subjects were instructed on where to stand, and for proper technique for performing the tests. They stood with the distal end of the first metatarsal directly in the centre of the mat. They then used the opposite foot to reach out as far as possible in the four directions. The distance that the farthest portion of their foot reached was recorded. The participants placed their hands on their iliac crests before starting and during the test. The entire stance foot remained on the ground throughout the entire test. Lifting part of the foot resulted in that portion of the test being re-done. Testing was conducted with both the dominant and the non-dominant leg as the stance leg. |
| Meszler and Vaczi, 2019 | Laboratory-based static balance test | Players stood on the stabilometer with one foot without shoes and kept the contralateral knee bent; the hips were level to the ground; the eyes were open and fixed on a spot marked on a screen. Visual feedback was continuously provided on the screen by displaying the magnitude of postural sway. Three trials were performed with both legs, with 1-min recovery between trials. The instrument aggregated the results of the test and evaluated the performance between 0 and 100 points. The value of the best trial was recorded for each leg, which was then averaged and used for statistical analyses |
| Myer et al., 2006 | Laboratory-based static balance test | The test comprised a single-leg hop forward (50 cm), performed three (randomized) times on each leg. A portable force platform was used. Subjects initiated the movement while balancing on one foot. They were instructed to hop forward 50 cm and balance for 10 seconds after the landing on the same foot. |
| Nobre et al., 2017 | Other dynamic balance test | The test was conducted using a standardised test for children developed in Germany for evaluating the gross motor coordination. This test involved walking backward on a balance beam 3 m in length, but of decreasing widths: 6 cm, 4.5 cm, 3 cm. For each task, performance was scored in a point system as suggested by the protocol, were summed and converted in the overall MQ gender and age specific. The overall MQ qualifies gross motor development in the following categories: ‘not possible’ (MQ<56), ‘severe motor disorder’ (MQ 56–70), ‘moderate motor disorder’ (MQ 71–85), ‘normal’ (MQ 86–115), ‘good’ (MQ 116–130) and ‘high’ (MQ131–145). |
| Piirainen et al., 2014 | Laboratory-based dynamic balance test | Dynamic balance was measured using a custom-made dynamic balance measurement system (University of Jyväskylä, Finland; HurLabs Oy, Tampere, Finland), which consists of four pneumatic cylinders placed vertically under a BT4 balance platform (HurLabs Oy, Tampere, Finland). Maximal cylinder displacement amplitude was 12.5 cm, giving 12° freedom of movement in each direction. By releasing air out of the cylinders, it was possible to drop (free fall) each side of the plate independently to produce a perturbation. During the measurements, subjects stood on the balance plate for two 30 s sets, during each of which four sudden balance perturbations were induced, one in each direction (medial–lateral, anterior–posterior). Subjects were unaware of the direction and timing of the perturbations. During each perturbation, one side of the plate dropped by 12.5 cm in free fall. A black mark was fixed on the wall 2.8 m from the subject at eye level to stabilize their visual focus during the measurements. For each perturbation direction, the attempt with the least-average centre of pressure sway was chosen for further analysis. |
| Porrati-Paladino and Cuesta-Barriuso, 2021 | Y-balance test | The subject stood in monopedal stance in the centre of an inverted Y-shape on the leg to be evaluated. Three attempts were made for each reaching direction (anterior, posteromedial, and posterolateral). The distance was measured in centimetres, and the arithmetic mean of three attempts made in each range was calculated. |
| Ramírez-Campillo et al., 2015a | Laboratory-based static balance test | On a balance platform (Bertec BP5050 balance plate platform; Bertec Corporation, Columbus, OH, USA; sample rate of 1,000 Hz), participants completed two tests (a) normal stance, eyes open, (b) normal stance, eyes closed. The average of two balance trials for each test was used for subsequent analysis and was expressed in centimetres. Both anterior-posterior and mediolateral data were collected during each trial. |
|  | Laboratory-based dynamic balance test | On a balance platform (Bertec BP5050 balance plate platform; Bertec Corporation, Columbus, OH, USA; sample rate of 1,000 Hz), participants completed two tests (a) perturbed stance, eyes open, and (b) perturbed stance, eyes closed. The perturbed stance condition was defined as that obtained while standing on a piece of foam 3 cm thick. The average of two balance trials for each test was used for subsequent analysis and was expressed in centimetres. Both anterior-posterior and mediolateral data were collected during each trial. |
| Ramírez-Campillo et al., 2015b | Laboratory-based static balance test | On a balance platform (Bertec BP5050 balance plate platform; Bertec, Corp., Columbus, OH, USA; sample rate of 1,000 Hz) participants completed two tests: (a) normal stance, eyes open and (b) normal stance, eyes closed. The average of two trials for each test was used for subsequent analysis. Both anteroposterior and mediolateral data were collected during each trial. |
| Ritzmann et al., 2018 | Laboratory-based static balance test | The control of posture was assessed in the monopedal stance with eyes open and eyes closed on a force plate (Leonardo Mechanography®, Novotec, Pforzheim, Germany) using the data acquisition unit Power1401-3 (CED, Cambridge, United Kingdom). The subjects stood barefoot in an upright position on their left leg, kept hands on their hips and directed their head and eyes forward. They were instructed to stand as still as possible, with the free leg not touching the other leg. Recordings were made twice in each condition over a period of ten seconds, separated by one-minute breaks; means were calculated. The displacement and velocity of the center of force, the dominant frequency and the standard ellipse area (90% movement area) was assessed using MATLAB® R2016a. The center of force displacement and standard ellipse area in medio-lateral and anterior- posterior direction were calculated. |
| Surakhamhaeng et al., 2020 | Laboratory-based static balance test | Standard deviations of Centre of Pressure in mediolateral and anteroposterior directions, and ranges of Centre of Pressure in mediolateral and anteroposterior directions were collected by using a force plate. The participants stood on their unstable foot or less severe Functional Ankle Instability foot on the force plate, arms crossed on the chest, eyes open and looking straightforward or eyes closed while standing still on their single leg for 20 seconds. To obtain reliable results, the participants performed two practice trials before the 3 real testing trials. |
|  | Laboratory-based static balance test | The time to stabilization of ground reaction force in mediolateral and anteroposterior directions were collected during the step-down test from a box 30 cm in height on the force plate. The mediolateral and anteroposterior time to stabilization were determined by sequential estimation. This technique involved an algorithm to calculate a cumulative average of the data points in series by successively adding one point at a time. The cumulative average was compared with the overall series mean. Stable series were considered when the sequential average remained within 0.25 SD of the overall series mean. The Time to Stabilization in vertical Ground Reaction Force was established as the time when the vertical force component reached and stayed within 5% of the subject’s body weight after landing. In addition, the markers were used to monitor the subject’s movement when they stepped down onto the force plate. |
|  | SEBT | Reach distances of a single leg in the anterior, posteromedial, and posterolateral directions were obtained from the modified SEBT. The participants stood on the unstable leg and used the other leg to move in each direction as far as possible. The absolute reach distance scores (cm) were averaged from 3 trials. Then, normalized reach distance (%) and composite reach distance scores (%) were computed with the use of the following formula: normalized reach distance (%) = absolute reach distance / leg length * 100 and composite reach distance (%) = sum of the 3 reach directions / 3 * leg length * 100 |
| Tay et al., 2019 | Flamingo test | Participants were asked to stand barefooted on one leg for 30 seconds with their eyes open. The arms were placed on the hips while the free leg was positioned next to the standing leg, with the knee flexed at 90° posteriorly (*flamingo* stand). Both legs were randomly tested for three trials. Centre of pressure trajectories during the balance tasks were sampled at 50 Hz using a force platform (model 9287BA, Kistler Instruments AG, Winterthur, Switzerland) and analysed using customized MATLAB codes. |
|  | Y-balance test | Participants stand on the platform on one leg. With toes behind the indicated line pushed the reach indicator using their free leg in the anterior, posteromedial, and posterolateral directions. Both legs were tested with the reach distances normalized to the limb length of the tested leg, measured from anterior superior iliac spine to medial malleolus. A composite score was also calculated as the average of the three normalized reach distances in different directions as an overall indication of dynamic balance performance. |
| Trecroci et al., 2015 | Y-balance test | The test involved a testing device to measure the distance reached in anterior, posteromedial and posterolateral direction while standing on each foot. Children performed the test barefoot to limit possible influences of footwear on balance. During all trials, participants were instructed to keep the hands on their hips and not to touch the floor with the reach foot except for marking their reach. The lower limb length was also measured to normalize the reach distance among participants. From a supine position, *true* leg length were obtained by measuring the distance between the anterior superior iliac spine and the medial malleolus. From these data, the composite reach score was calculated by summing the maximum reach distance for the three reach directions on a given limb and dividing by three times the limb length prior to multiplying by 100 to reference the composite reach as a percentage of leg length. |
| Witzke and Snow, 2000 | Laboratory-based static balance test | Static balance was measured using the Biodex Stabilometer (Shirley, NY). Two 30-s trials using a stability level of 2 (range 1–8, 1 being least stable/most difficult and 8 being most stable/least difficult) was performed. The same foot position was used for pre- and post-tests. |
| **Abbreviations:** BESS: balance error scoring system; ICC: intraclass correlation coefficient; MQ: motor quotient; SD: standard deviation SEBT: star excursion balance test; TTS: time to stabilization. | | |
